# Plant-derived extracellular vesicles for drug delivery: current and future

**DOI:** 10.1093/rb/rbag109

**Published:** 2026-06-02

**Authors:** Fengdan Xu, Yuling Liu, Qiling Zhang, Ruying Tang, Hui Li, Hongjun Yang, Longfei Lin

**Affiliations:** State Key Laboratory for Quality Ensurance and Sustainable Use of Dao-di Herbs, Institute of Chinese Materia Medica, China Academy of Chinese Medical Sciences, Beijing 100700, China; State Key Laboratory for Quality Ensurance and Sustainable Use of Dao-di Herbs, Institute of Chinese Materia Medica, China Academy of Chinese Medical Sciences, Beijing 100700, China; State Key Laboratory for Quality Ensurance and Sustainable Use of Dao-di Herbs, Institute of Chinese Materia Medica, China Academy of Chinese Medical Sciences, Beijing 100700, China; State Key Laboratory for Quality Ensurance and Sustainable Use of Dao-di Herbs, Institute of Chinese Materia Medica, China Academy of Chinese Medical Sciences, Beijing 100700, China; State Key Laboratory for Quality Ensurance and Sustainable Use of Dao-di Herbs, Institute of Chinese Materia Medica, China Academy of Chinese Medical Sciences, Beijing 100700, China; Institute of Traditional Chinese Medicine Health Industry, China Academy of Chinese Medical Sciences, Nanchang 330000, China; China Academy of Chinese Medical Sciences, Beijing 100700, China; State Key Laboratory for Quality Ensurance and Sustainable Use of Dao-di Herbs, Institute of Chinese Materia Medica, China Academy of Chinese Medical Sciences, Beijing 100700, China

**Keywords:** plant-derived extracellular vesicles, drug delivery systems, loading methods, challenges and solutions, development trends

## Abstract

Plant-derived extracellular vesicles (PDEVs) have attracted considerable attention as natural drug delivery vehicles owing to their low immunogenicity, excellent biocompatibility, cross-kingdom delivery capability and intrinsic targeting properties. They naturally encapsulate a variety of bioactive components that can synergize with loaded drugs, while the vesicles exhibit good stability under simulated gastrointestinal conditions. This review focuses on the structure-property-function relationships of PDEVs in drug delivery. It systematically compares current drug loading strategies and evaluation approaches, particularly engineered loading technologies and composite delivery systems. Furthermore, it summarizes the applications of PDEV-based delivery systems in disease therapy, vaccine development, cosmetics and nutraceuticals. Finally, we propose an evaluation framework to facilitate clinical translation, providing theoretical support for advancing these systems toward practical use.

## Graphical abstract

**Graphical Abstract:**
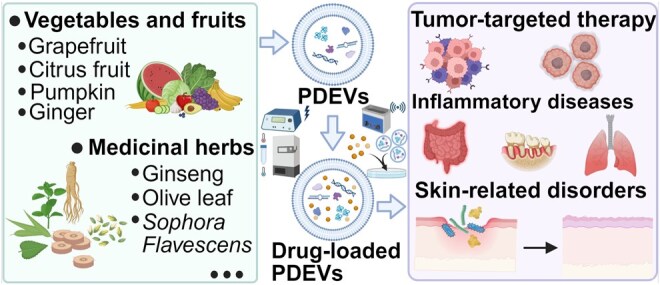

## Introduction

Nanomedicine-based drug delivery systems (NDDSs) represent a prominent area of research in contemporary drug development and therapeutic applications, demonstrating considerable promise. The nanocarriers employed for drug delivery primarily include liposomes, polymeric nanoparticles, inorganic nanomaterials (such as gold nanoparticles and iron oxide nanoparticles), carbon nanomaterials, dendrimers and protein-based nanoparticles [1, 2]. NDDSs can enhance therapeutic efficacy through targeted delivery, controlled release kinetics and protection of therapeutic agents from premature degradation. However, their clinical translation is still hindered by challenges including toxicity concerns, poor biodegradability and complex manufacturing processes [1]. In recent years, extracellular vesicles have emerged as a significant focus in the biomedical field due to their inherent properties as natural drug delivery carriers, unique biological characteristics and favorable biocompatibility [3–5]. Extracellular vesicles, which are abundantly derived from animals, plants and microorganisms, offer distinct advantages over other nanocarriers, including low immunogenicity, inherent targeting capabilities, biodegradability and natural homing abilities. These properties enable them to efficiently traverse biological barriers and facilitate precise drug delivery, thereby enhancing therapeutic efficacy [3]. Additionally, engineered modifications of extracellular vesicles have significantly improved drug-loading efficiency, targeting precision and resistance to bodily clearance, thereby substantially expanding their clinical application potential and therapeutic scope [4]. Current research predominantly focuses on animal-derived extracellular vesicles (ADEVs), with extensive studies on their biological functions, molecular mechanisms and translational applications. In contrast, research on plant-derived extracellular vesicles (PDEVs) remains relatively underdeveloped, lagging behind both in mechanistic understanding and clinical translation efforts. Nevertheless, accumulating evidence suggests that PDEVs offer distinct advantages in traversing species barriers to deliver bioactive molecules, positioning them as promising candidates for next-generation biotherapeutic and drug delivery nanoplatforms [6].

In comparison with ADEVs, PDEVs tend to exhibit a broader size distribution and greater structural heterogeneity, making them ideal natural nanocarriers for enriching diverse bioactive constituents specific to their parent plants [7]. For example, blueberry-derived PDEVs are enriched in miR166 and miR396 families. Target gene prediction results indicate that these miRNAs potentially regulate pathways associated with the human digestive system, immune system and infectious diseases [8]. PDEVs isolated from edible plant juices contain miR156a-5p, miR166a-3p and miR168a-5p, with fluorescence labeling confirming their uptake by rat intestinal cells [9]. In addition to miRNAs, the proteins, lipids and metabolites encapsulated in PDEVs also possess various biological functions, making them promising candidates for natural nanomedicines [10]. PDEV-based drug delivery systems possess significant potential in achieving synergistic therapeutic effects, which arise from the combination of their endogenous components and the encapsulated drugs [11–15]. The therapeutic advantages of this synergy are multifaceted, ranging from enhanced therapeutic efficacy and reduced systemic toxicity to improved targeting ability, reversal of drug resistance and enhanced drug stability [14]. Upon loading with the chemotherapeutic agent doxorubicin, ginger-derived extracellular vesicles (GDEVs) demonstrated pH-responsive drug release superior to that of conventional liposomal formulations [11]. This feature enables selective drug release in the acidic tumor microenvironment, leading to enhanced antitumor effects with concomitantly reduced toxicity toward healthy tissues. Although still limited in number, clinical trials of PDEVs as therapeutic carriers have completed Phase I, such as GDEVs loaded with curcumin for the treatment of irritable bowel syndrome [16].

Recent years have witnessed a burgeoning number of studies on PDEVs. Comprehensive reviews have extensively discussed their molecular composition [13, 17–19], biological functions [10, 20], isolation and characterization methods [13, 18, 20], as well as their therapeutic roles in various disease models [15, 19, 21]. Building on this foundation, these systems have attracted considerable attention as drug delivery platforms, with applications explored in areas including cancer, inflammatory diseases, tissue repair and intestinal homeostasis regulation [12, 19, 22]. Furthermore, progress in drug loading strategies [13, 19], engineering modifications [23] and strategies for quality control and nonclinical evaluation [24, 25] has been systematically reviewed. In functional foods and cosmetics, PDEVs present unique advantages for improving the bioavailability and targeted delivery of poorly soluble active ingredients, enabling the development of high-efficacy nutritional supplements and advanced transdermal delivery systems [26–29]. However, relatively little effort has been devoted to summarizing the structure–function relationships of PDEV-based drug delivery systems, as well as PDEV-integrated delivery systems.

This review focuses on the construction of PDEV-based drug delivery systems, correlating their physicochemical and structural characteristics with drug delivery performance. It systematically compares diverse loading strategies and evaluation criteria and further proposes a framework for the development of assessment strategies toward clinical translation. Importantly, it highlights engineered and composite PDEV-based drug delivery systems, with a particular emphasis on evaluating their application prospects and future directions in tissue repair and regenerative biomaterials.

## Characteristics and advantages of PDEVs

### Physical properties

PDEVs are bilayer membrane-structured nanovesicles released by plant cells, typically appearing spherical or cup-shaped under electron microscopy [30, 31]. Their particle size ranges from 50 to 500 nm. The size and surface charge of PDEVs vary depending on plant species, cultivation methods and extraction protocols. For example, ultracentrifugation (UC) of sweet orange juice isolates two distinct vesicle populations: a smaller vesicle group averaging 62 ± 12 nm in diameter and the largest vesicles measuring 247 ± 61 nm [32]. PDEVs isolated from *citrus clementina* fruit juice via differential centrifugation and density gradient centrifugation exhibit a size distribution ranging from 75 nm to 345 nm [33]. PDEVs isolated from *Arabidopsis thaliana* callus tissue and apoplastic fluid show sizes of 222.8 ± 36.5 nm and 283.6 ± 58.3 nm, respectively [34]. However, some studies have reported larger dimensions. For instance, PDEVs isolated from 11 distinct edible fruits and vegetables via differential centrifugation range from 100 to 1000 nm in size [35]. The apparent size increase likely stems from nanoparticle aggregation in the samples—a phenomenon amplified by dynamic light scattering due to its hypersensitivity to size variations, where scattered light intensity scales with the sixth power of particle diameter [32]. Zeta potential serves as a crucial indicator for evaluating the stability of colloidal systems, reflecting the electrostatic repulsion capacity of PDEVs against aggregation and their stability in physiological environments. PDEVs typically carry negative surface charges, with zeta potentials ranging from –10 to –50 mV. For example, PDEVs derived from *Curcuma longa* exhibit a zeta potential of –17.6 ± 1.19 mV [36]. In another study, the zeta potentials of PDEVs isolated from *Arabidopsis thaliana* callus and apoplastic fluid were −23.8 ± 1.3 mV and −30.5 ± 2.2 mV, respectively [34]. The particle size, shape and zeta potential of nanoparticles are critical determinants of drug delivery efficiency and *in vivo* performance. Spherical particles within the 10–200 nm size range and with a zeta potential exceeding ± 10 mV demonstrate enhanced drug delivery efficacy [37]. This suggests that PDEVs are natural drug delivery carriers.

### Chemical composition

PDEVs contain a variety of bioactive compounds, including small RNAs (e.g. miRNAs, siRNAs and piRNAs), proteins (e.g. heat shock proteins, annexins, chloroplast proteins and cell wall-related proteins), lipids (e.g. phosphatidic acid and phosphatidylcholine) and various metabolites (e.g. carotenoids, flavonoids, vitamin C and polyphenols) [10, 20]. Small RNAs are important regulatory molecules in plants, specifically sorted into PDEVs through RNA-binding proteins, including Argonaute 1, RNA helicases and annexins [38]. Small RNAs, particularly miRNAs, not only mediate intra- and inter-species communication in plants but also transfer across plant-microbe, -insect and -mammalian systems, achieving cross-kingdom regulation [39]. Rice-derived *MIR168a* can be ingested through the diet and enter the bodies of mice and humans, where it targets and suppresses the expression of the murine hepatic gene *LDLRAP1*, thereby impairing low-density lipoprotein clearance [40]. This provides direct evidence for the cross-kingdom regulation of mammalian physiological functions by plant miRNAs. However, such regulation remains controversial, as independent studies have failed to reproduce the original findings [41]. Nevertheless, statistical analysis of large-scale cancer genomic data demonstrates that specific plant miRNAs exhibit high sequence complementarity to under-expressed human miRNAs, suggesting their potential to function as surrogate molecules in regulating human gene expression [42]. A notable feature of plant miRNAs is the 2'-O-methylation at their 3' terminus, which confers stability in the complex digestive environment and during systemic circulation, thus providing a natural advantage for their use in oral drug delivery [21].

In terms of protein composition, PDEVs are enriched in annexins, heat shock proteins, aquaporins and other functional proteins. These proteins play crucial roles in maintaining the structural stability of the PDEV membrane, facilitating substance transport and mediating signal transduction [10, 18, 20, 30, 43]. Proteomic analysis revealed that PDEVs carry various cell wall-hydrolyzing enzymes (e.g. 1,3-β-glucosidase, pectinesterase), which can assist their passage through the plant cell wall and facilitate substance transport [44]. Defense-related proteins such as rpm1-interacting protein 4 and their interacting partners mediate immune signaling transduction. Phospholipase Dα and phospholipase Dδ regulate reactive oxygen species signaling and membrane trafficking proteins, including penetration1 (PEN1), syntaxin of plants 122 (SYP122), SYP132, Patellin1 and Patellin2, participate in vesicle transport and secretion [45]. Furthermore, certain proteins mediate cargo sorting in PDEVs. For instance, RNA-binding proteins facilitate the sorting of small RNAs [38] and the tetraspanin 8 (TET8) acts as a transporter for glycosylinositol phosphoceramide (GIPC), regulating the maturation of GIPC-enriched multivesicular bodies (MVBs) and the secretion of extracellular vesicles [46].

The lipid composition notably serves to set PDEVs apart from ADEVs. Lipidomic analysis identified six principal lipid classes in PDEVs from four species: sphingolipids, glycerolipids, glycerophospholipids, sterol lipids, fatty acyls and prenol lipids [47]. Sphingolipids in PDEVs are predominantly composed of GIPCs. In *Arabidopsis* leaf-derived PDEVs, GIPCs constitute approximately 99% of the total sphingolipids, demonstrating a specific enrichment compared with leaf tissues (about 60%) [48]. Glycerolipids include digalactosyldiacylglycerol and monogalactosyldiacylglycerol, while glycerophospholipids comprise phosphatidic acid, phosphatidylcholine, phosphatidylethanolamine and phosphatidylinositol [19]. The compositional proportions of glycerolipids and glycerophospholipids vary significantly among PDEVs from different sources [19, 44, 47]. For instance, in PDEVs derived from grapefruit, the content of glycerophospholipids reaches 50.05%, whereas in those from ginger, lemon and grape, the glycerolipid content (46.23%, 53.77% and 47.97%, respectively) is significantly higher than that of glycerophospholipids (less than 30%) [47]. It has been proposed that the differences in lipid composition of PDEVs contribute to their specific targeting of specific cells or organs [44]. While direct evidence linking the lipid profile of PDEVs to cellular targeting remains limited, substantial evidence from ADEVs research demonstrates that their lipid composition plays a crucial role in mediating specific recognition and signal transduction [49]. In contrast to ADEVs, PDEVs do not contain cholesterol. Instead, this function is substituted by phytosterols (e.g. β-sitosterol and stigmasterol), which serve as structural analogues [50, 51]. The content of β-sitosterol modulates vesicle characteristics and drug-loading stability. A study on liposomes revealed that incorporating β-sitosterol at a concentration of 20–33 mol% effectively reduced vesicle size, improved distribution and significantly enhanced the encapsulation efficiency, storage stability and bioavailability of curcumin [52].

Another notable characteristic of PDEVs is their abundance of secondary metabolites derived from parent plants. These components typically possess antioxidant activity, such as anthocyanins [53], curcumin [54], trans-δ-viniferin [55] and vincristine [56]. The diverse secondary metabolites endow PDEVs with a broad spectrum of pharmacological activities. When employed for drug delivery, these endogenous active metabolites can exert synergistic effects with the loaded drug molecules, thereby enhancing therapeutic efficacy [14]. Notably, the accumulation of plant secondary metabolites in PDEVs is passive, driven by their physicochemical properties such as lipophilicity, rather than active packaging [44]. This implies that PDEVs possess a natural packaging propensity for lipophilic drugs, providing a theoretical rationale for designing PDEV-based delivery systems.

### Biogenesis and biological function

**Figure 1:**
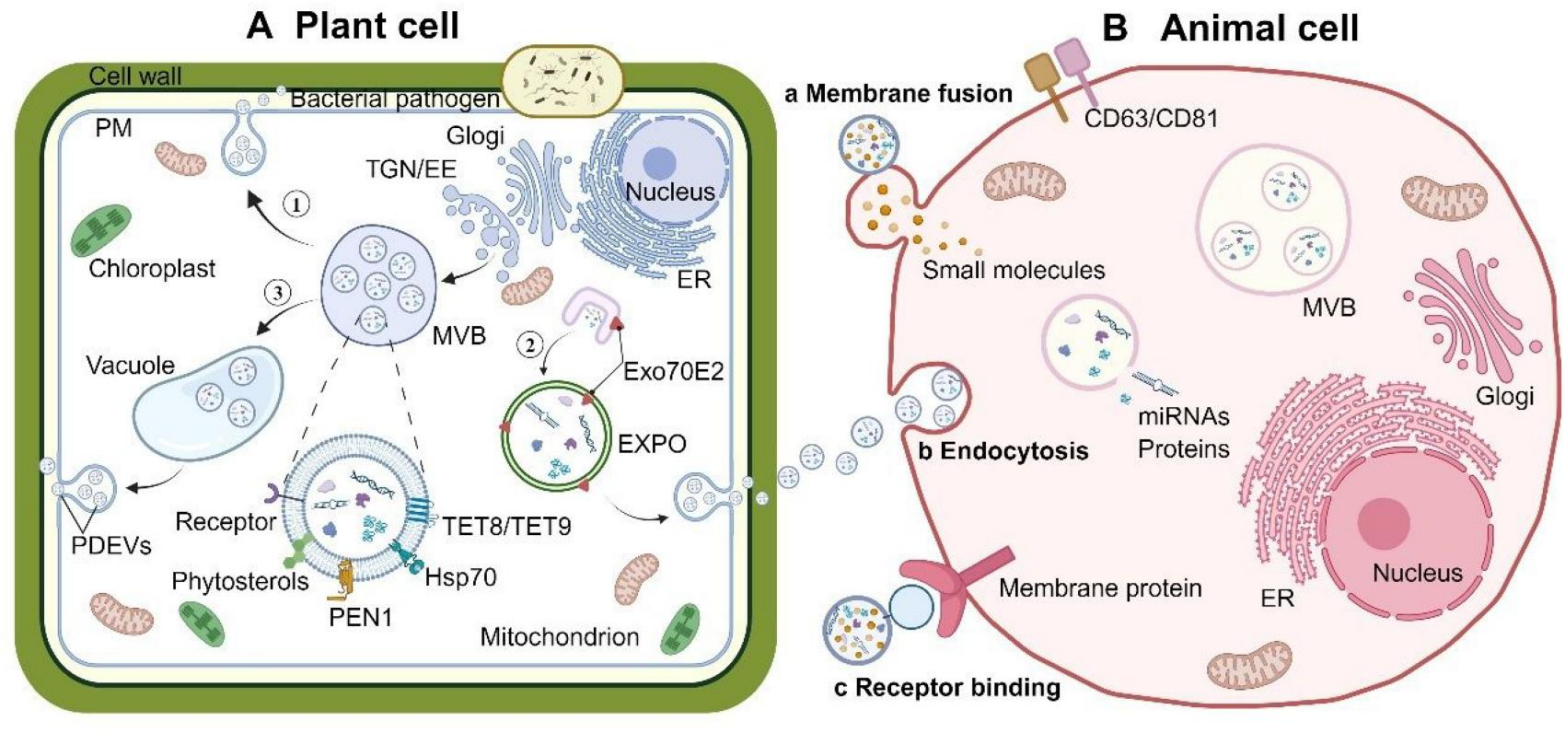
Biogenesis and cellular uptake of plant-derived extracellular vesicles. (**A**) When plants are attacked by pathogens, they release defensive plant-derived extracellular vesicles (PDEVs) through multiple pathways, including the multivesicular body (MVB) pathway, the exocyst-positive organelle (EXPO) pathway and the vacuolar pathway. MVBs, formed through endocytosis as late endosomes, can directly fuse with the plasma membrane (PM) to release their intraluminal vesicles (ILVs) into the extracellular space as PDEVs. Alternatively, they can transport ILVs to the vacuole, enabling the release of PDEVs through fusion of the vacuolar membrane with the PM. EXPO, a novel organelle characterized by a spherical double-membrane structure, discharges PDEVs carrying the exocyst subunit Exo70 upon fusion with the PM. (**B**) PDEVs can be taken up by animal cells through three mechanisms: (a) membrane fusion; (b) endocytosis; (c) receptor–ligand binding. PDEVs deliver diverse functional molecules, including nucleic acids, proteins and small-molecule drugs, into animal cells to regulate physiological pathways. Created with BioRender.com.

PDEVs serve as key mediators in diverse biological processes—including plant growth and development, material transport, signal transduction and immune regulation—establishing them as essential functional vehicles in plant biology. It is currently believed that the biogenesis of PDEVs primarily relies on three mechanisms: the MVB pathway, the exocyst-positive organelle (EXPO) pathway and the vacuole pathway [30], as illustrated in Figure 1A. The MVB pathway depends on the endosomal sorting complex required for transport (ESCRT), including ESCRT-I, ESCRT-II and ESCRT-III, and plasma membrane-associated proteins (such as TET8 and PEN1). In this progress, ESCRT complexes recognize ubiquitinated proteins, promote membrane deformation and regulate MVB maturation, whereas plasma membrane-associated proteins contribute to PDEVs release [34]. The EXPO pathway serves as an unconventional protein secretion mechanism that delivers certain signal peptide-lacking cytosolic proteins, such as XTH29 (a specific member of the xyloglucan endotransglucosylase/hydrolase family), to the cell wall [57, 58]. The MVB pathway generates PEN1- and TET8-positive PDEVs [59], whereas the EXPO pathway produces Exo70B2-positive PDEVs [60]. Notably, TET8-positive PDEVs are enriched in GIPCs [48]. Characterized by long-chain fatty acids and complex glycosylated head groups, GIPCs interact with plant sterols such as β-sitosterol to promote the formation of liquid-ordered domains. These sterol-sphingolipid interactions contribute to plasma membrane stability, correlating with increased membrane thickness, while the negatively charged glycan heads of GIPCs further enhance membrane surface charge [61]. This ordered structure, arising from sterol-sphingolipid interactions, enhances hydrophobic packing within the membrane [61, 62], creating a suitable microenvironment to accommodate lipophilic drugs. Meanwhile, the negatively charged glycan heads of GIPCs extend into the aqueous exterior [61], providing potential binding sites for cationic drugs through electrostatic adsorption, thus suggesting a dual-mode loading strategy. PEN1-positive PDEVs partially co-localize with PEN3, an ATP-binding cassette transporter, and show significantly increased secretion during the early stages of fungal infection [59]. These features suggest their potential as naturally occurring vesicles that may carry antifungal components to fungal surfaces during infection. However, the potential of Exo70B2-positive PDEVs in drug delivery remains poorly understood.

Although its specific cargo loading mechanism remains unclear, the vacuolar pathway is activated during plant effector-triggered immunity, relying on vacuolar sorting receptors to mediate tonoplast-plasma membrane fusion, thereby releasing hydrolytic enzymes and other contents to execute defensive functions [63]. Additionally, the autophagic pathway has been suggested in the biogenesis of PDEVs. Autophagosomes can fuse with MVBs to form amphisomes, which subsequently fuse with the plasma membrane to release their contents [17, 64, 65]. When subjected to biotic or abiotic stress, plants release increased PDEVs [59, 66–68]. These PDEVs exhibit a distinct, stress-specific molecular composition, thus enabling active defense and immunomodulation. For instance, during aphid infestation, melon phloem vesicles accumulate active proteasomes and defense proteins, thereby enhancing insect resistance [66]. *Arabidopsis thaliana* produces distinct extracellular vesicle subpopulations, such as TET8^+^ and PEN1^+^, which respond to fungal infection and hormonal cues like salicylic acid by directly interacting with pathogens and altering their development to delay infection [59]. Furthermore, PDEVs contribute to cell wall remodeling through the targeted transport of precursors and key enzymes for lignin synthesis to the cell wall [69].

PDEVs can be taken up by animal cells through multiple pathways and regulate animal physiological processes via the bioactive compounds they carry. The internalization of PDEVs is a complex process involving various mechanisms such as endocytosis, membrane fusion and receptor-ligand recognition, as illustrated in Figure 1B. Within living organisms, PDEVs demonstrate multiple therapeutic benefits, including antitumor, antioxidant and tissue regeneration-promoting, as well as the regulation of metabolism and immunity [70]. Multiple studies have identified miRNAs as key components through which PDEVs exert their diverse biological effects [42, 71]. Plant-derived miRNAs assemble with the mammalian Argonaute 2 protein to form the RNA-induced silencing complex. Depending on the degree of sequence complementarity, these complexes mediate either target mRNA cleavage or translational repression, thereby enabling cross-kingdom regulation of gene expression in mammals [21]. Recent research demonstrates PDEVs from *Polygoni Multiflori Radix* utilize caveolin- and heparan-sulfate-proteoglycan-dependent endocytosis to enter human dermal papilla cells, where plant-derived miRNAs directly silence the human androgen receptor, exemplifying cross-kingdom gene regulation [72]. The lipids and proteins in PDEVs act as functional components and mediate cross-kingdom regulation by participating in cargo sorting, targeted delivery and cellular uptake. This uptake into intestinal epithelial cells via macropinocytosis and endophilin-mediated endocytosis, a process dependent on the PDEVs’ lipid and protein components [73]. Further underscoring the role of specific surface molecules, another study showed that garlic-derived PDEVs are internalized by animal hepatocytes through a lectin II-mannose interaction with host cluster of differentiation 98 (CD98) [74]. Additionally, PDEVs enhance the antioxidant capacity of human cells by triggering intracellular calcium ion release and activating downstream signaling, unveiling a plant-to-human route of cross-kingdom regulation [75].

### Inherent advantages

Compared to ADEVs, PDEVs are derived from a far wider range of sources. To date, PDEVs have been successfully isolated from diverse plant species, including common fruits and vegetables (e.g. grape, ginger) as well as medicinal plants with specific pharmacological value (e.g. ginseng, *Salvia miltiorrhiza*) [76–79]. The diverse plant origins endow PDEVs with a wide spectrum of biological functions. Grape-derived PDEVs promote intestinal epithelial regeneration via stem cell signaling pathway activation [77], while ginseng-derived PDEVs exhibit anti-inflammatory and immunomodulatory effects by regulating immune-related pathways [76]. The abundant availability of plant resources positions PDEVs favorably for large-scale production and industrial application [18, 20, 80, 81]. PDEVs demonstrate superior productivity under identical processing conditions. For example, isolation and concentration of PDEVs from 50 mL of grapefruit juice yielded 1 × 10^12^ particles mL^−1^ (600 µL), corresponding to an estimated total of 6 × 10^11^ particles—approximately 300-fold the yield of ADEVs recovered from an equivalent volume of culture medium [82]. Furthermore, the introduction of plant cell culture technology provides a feasible solution for actively controlling batch-to-batch consistency of PDEVs at the production source. Compared with animal cell culture, plant cell culture does not require complex serum additives, thereby avoiding ethical concerns and biological contamination risks, which facilitates the development of a more stable production process [17, 24]. In terms of safety, PDEVs are free from zoonotic viruses and have a lower likelihood of inducing hypersensitivity reactions [6]. PDEVs exhibit remarkable physicochemical stability and resistance to gastrointestinal digestion, providing an important foundation for their stable drug delivery in complex physiological environments. This stability is exemplified by olive-derived PDEVs, which retained their size and yield following exposure to high temperature (70°C, 1 h), a broad pH range (5–10) and repeated extrusion through 50–100 nm pores [83]. Similar stability has been observed in ginger-derived PDEVs, which maintained their particle size and zeta potential after incubation in simulated gastric fluid (pH 2.0, containing pepsin) and simulated intestinal fluid (pH 6.5, containing bile and pancreatin) [84].

In contrast to artificially synthesized liposomes and polymeric nanoparticles, PDEVs benefit from their natural origin, which endows them with excellent biocompatibility and low immunogenicity [18]. These inherent properties also translate into superior drug-loading stability, cellular uptake and targeting capacity. For example, Jiang *et al*. [85] encapsulated luteolin in PDEVs derived from sesame leaves (Exo@Lu) and evaluated stability under various external factors (including light, heat and pH). Exo@Lu retained 91.9% of luteolin after 1 h at 60°C and 70.7% after 7 h of UV exposure, versus 37% and 25% for free luteolin, respectively. Further expanding this versatility, GDEVs exemplify how PDEVs can mediate complex therapeutic actions. Enriched in phosphatidic acid, GDEVs are internalized by intestinal Caco-2 cells through caveolar endocytosis and macropinocytosis [86]. Beyond delivering endogenous anti-inflammatory miRNAs, GDEVs act as stable nanocarriers for exogenous cargos such as TNF-α (tumor necrosis factor-alpha) siRNA. Their membrane integrity and RNA payload are both maintained intact after exposure to simulated gastric and intestinal fluids, enabling a combined strategy of gene silencing and intrinsic anti-inflammation [87]. A multidimensional comparison of PDEVs, ADEVs and liposomes is included in Table 1.

**Table 1:** Comparison of plant-derived extracellular vesicles, animal-derived extracellular vesicles and liposomes.

Aspect	Plant-derived extracellular vesicles	Animal-derived extracellular vesicles	Liposomes	References
Biogenesis	Primarily via the MVB pathway; plant-specific routes such as EXPO and vacuolar pathway have also been reported, though their prevalence and functional significance remain to be clarified	Primarily MVB pathway and plasma membrane budding	NA	[17, 88]
Structure and morphology	Structure: phospholipid bilayer Size: 30–500 nm, depending on source and isolation method Shape: spherical, cup‑shaped or ellipsoidal (influenced by dehydration during sample preparation)	Structure: phospholipid bilayer Size: 30–150 nm (exosomes); microvesicles can range up to 1000 nm Shape: spherical or ellipsoidal	Structure: phospholipid bilayer Size: tunable by preparation method Shape: generally spherical or ellipsoidal vesicles	[7, 89]
Membrane composition	Rich in plant‑specific lipids such as phytosterols, GIPCs, composition varies significantly with source	Rich in cholesterol, sphingomyelin and phosphatidylserine	Composed primarily of natural or synthetic phospholipids, often with cholesterol to enhance stability	[44, 88, 89]
Cargo	Lipids, proteins, nucleic acids (e.g., miRNA, siRNA), plant secondary metabolites	Lipids, proteins, nucleic acids, cytokines, growth factors and other bioactive molecules	No intrinsic cargo; capable of drug loading	[44, 88, 89]
Biological activity	Exhibits anti‑inflammatory, antioxidant, tissue‑regenerative and other activities in various *in vitro* and animal models	Involved in physiological and pathological processes including immune regulation, intercellular communication and tissue regeneration	Generally lack intrinsic biological activity, although cationic lipids (e.g., DOTAP) may exhibit adjuvant activity and fusogenic lipids (e.g., DOPE) can facilitate membrane fusion	[7, 89]
Immunogenicity	Displays low immunogenicity in xenogeneic models, although the immunological risks associated with repeated administration or specific routes of delivery require systematic evaluation	Immunogenicity depends on the source (autologous, allogeneic, xenogeneic); xenogeneic sources carry a risk of immune rejection, whereas allogeneic sources pose a moderate risk	Generally low immunogenicity, though cationic or PEGylated liposomes may trigger complement activation and other immune‑related responses	[7, 13, 89, 90]
Targeting capability	Some studies suggest that plant‑specific surface molecules (e.g., GIPCs, lectin‑like proteins) mediate interactions with mammalian cells, but the *in vivo* targeting mechanisms remain to be elucidated	Exhibits tissue tropism and can achieve targeting via surface ligand–receptor interactions; tissue‑specific targeting often requires engineering	Lack intrinsic active targeting capability; active targeting requires surface modification (e.g., ligand conjugation, antibody modification)	[48, 74, 88]
Stability	Generally exhibit good stability, particularly in lyophilized form, though stability varies with source, isolation method and storage conditions	Susceptible to degradation under ambient conditions or repeated freeze–thaw cycles; stability can be improved by lyophilization or the addition of cryoprotectants	Good stability; can be lyophilized for long‑term storage	[91–93]
Safety	No known risk of zoonotic pathogen transmission, although safety considerations apply if derived from genetically modified plants or contaminated raw materials	Depends on donor source, purification process and the risk of pathogen contamination	Safety profile is controllable, but attention should be paid to potential side effects such as complement activation and cytotoxicity associated with cationic liposomes	[7, 89]
Production cost	Raw materials are widely available, but yield and purification efficiency vary considerably with plant species, tissue type and extraction method	High cost, due to cell culture, specialized purification equipment and low yield	Moderate cost, with well‑established and controllable manufacturing processes	[12, 13]
Ethical concerns	Minimal	Involve ethical and biosafety issues related to donor cell sources	Minimal	[18]
Application areas	Functional foods, cosmetics, drug delivery systems	Primarily in drug delivery, diagnostics and regenerative medicine	Drug delivery, vaccine adjuvants, cosmetics	[18, 89]
Clinical progress	In early‑stage clinical exploration	Multiple ongoing clinical trials	Marketed	[16]

Abbreviations: DOPE, 1,2-dioleoyl-sn-glycero-3-phosphoethanolamine; DOTAP, 1,2-dioleoyl-3-trimethylammonium-propane; GIPCs, glycosylinositol phosphoceramide; MVB, multivesicular bodies; PEG: polyethylene glycol.

### PDEVs from different plant sources

PDEVs derived from different plant sources exhibit significant variations in physical properties, chemical composition and biological functions (Table 2). Given the ready availability of *Citrus* species, they have been the most extensively studied. *Citrus*-derived PDEVs are rich in flavonoids (e.g. naringin, neohesperidin) and vitamin C, making them ideal candidates for nutraceutical applications and drug delivery carriers [94, 95]. Ginger-derived PDEVs are enriched in gingerols and shogaols and exhibit natural colon-targeting properties, with distinct nanoparticle subpopulations displaying differential anti-inflammatory efficacy, underscoring the functional heterogeneity that necessitates subpopulation-specific isolation [84]. PDEVs from medicinal plants harbor unique secondary metabolites and miRNAs that confer distinctive pharmacological activities, exemplified by ginsenosides in ginseng [76], miR-115-5p in *Rhodiola rosea* [96], miR-828b in tea leaf [97] and bioactive constituents in *Salvia miltiorrhiza* with unclarified chemical identities [79]. However, interspecies variability, varietal differences and vesicle heterogeneity pose challenges to batch-to-batch consistency, and the dissection of functional subpopulations remains a critical priority for translational development.

**Table 2:** Comparison of plant-derived extracellular vesicles from different sources.

Plant source	Physical properties	Chemical composition	Biological function	Advantages	Disadvantages	References
*Citrus clementina*	Size: 103.3 ± 52.3 nm	Proteomic analysis identified 1018 proteins, including the marker proteins clathrin heavy chain and patellin-3-like protein	NA	High yield (1.16 × 10¹² particles/L) Rich protein composition	Marked vesicle heterogeneity	[33]
Red mandarin (*Citrus reticulata* Blanco cv. ‘Dahongpao’)	Average size: 190 nm Zeta potential: –5 mV	Phenolics and flavonoids, including neohesperidin, sinensetin and nobiletin	Antioxidant and anti-inflammatory activities Promising drug delivery carrier	Oxidative damage mitigation in inflammatory disorders Biocompatible natural carrier	Remarkable stability loss following 15 days of storage at 4°C	[94]
*Citrus limon*	Average size: 153.8 nm	Main lipids: capric acid (23%), tridecylic acid (28%) and lauric acid (21%)	Downregulated chondrogenic differentiation	Efficient uptake by human adipose‑derived stem cells	Potentially unsuitable for cartilage regeneration	[99]
*Citrus limon L.*	Average size: 100 nm	NA	Anticancer	Uptake by triple-negative breast cancer cells Synergy with DOX for enhanced efficacy and dose reduction	Unclear synergy mechanism Insufficient *in vivo* validation	[100]
*Citrus × paradisi*	Size: 210.8 ± 48.62 nm Zeta potential: ranging from –49.2 to –1.52 mV	Enriched with PE (45.52%) and PC (28.53%) Naringin and its metabolite naringenin	Anti‑inflammatory and immunomodulatory Promising drug delivery carrier	Intestinal macrophage targeting, high gastrointestinal stability and excellent oral safety	Marked vesicle heterogeneity Key functional factors unknown	[95]
Grapefruit	Size: 170.9 ± 5.7 nm Zeta potential: –20.16 ± 1.07 mV	Lipid composition similar to that of human primary astrocyte-derived exosomes	Antitumor	High yield, feasible surface modification and negligible cytotoxicity	No available drug loading and release data Absence of *in vivo* verification	[82]
Ginger	Average size: 231.6 nm Zeta potential: –12 mV	Main lipids: PA, DGDG and MGDG Low protein content 125 contained miRNAs (15–27 nucleotides) Active small molecules: 6‑gingerol and 6‑shogaol	Anti‑inflammatory and pro‑healing effects, with good efficacy in multiple colitis models	High yield Colon‑targeted Excellent gastrointestinal stability	Functionally heterogeneous subpopulations requiring precise separation Complex mechanisms and largely uncharacterized miRNA functions	[84]
Ginger	Size: 156 ± 36 nm Zeta potential: –26.6 ± 5 mV	Main miRNAs: bdi-miR5179, csi-miR396e-5p and others	Anti‑inflammatory	Potential to modulate human inflammatory and oncogenic pathways Well-defined intestinal cell uptake mechanism	Lack of *in vivo* functional validation	[86]
*Panax ginseng* C.A. Mey (Araliaceae)	Average size: 344.8 nm Zeta potential: –25.4 mV	Enriched with DGMG, PE and ceramide Diverse metabolites (e.g., amino acids, nucleotides) Highly enriched in ginsenoside Rg3	Antioxidant and antitumor	High yield Proven efficacy Excellent *in vivo* stability	Only validated in the B16F10 model Broad applicability requires further evaluation	[76]
*Salvia miltiorrhiza*	Average size: 105.15 nm Zeta potential: –25.2 mV	NA	Pro-angiogenic	High yield and safety Stable at −80 °C for one month	Unclear active molecules and mechanisms	[79]
*Rhodiola rosea*	Size: 30 − 200 nm Zeta potential: –11.1 mV	Secondary metabolites mainly including phenols and derivatives, flavonoids, alkaloids Multiple miRNAs, including the novel miRNA-115-5p	Antioxidant, anti-pyroptotic and pro-angiogenic	Well-defined therapeutic potential and excellent safety profile in lower limb ischemia	Lack of pharmacokinetic studies and long-term toxicity evaluation	[96]
*Camellia sinensis*	Size: 123.2 nm Zeta potential: ranging from –95 to –25 mV	Multiple secondary metabolites (e.g., flavonoids, phenolic acids, alkaloids)	Antioxidant, anti-melanogenic, anti-inflammatory and pro-autophagic	Excellent transdermal permeability Multifunctional bioactivity, superior efficacy to conventional extracts and low cytotoxicity	Unvalidated in normal human epidermal melanocytes Insufficient data on normal cell selectivity and safety	[97]

Abbreviations: DGDG, digalactosyldiacylglycerol; DGMG, digalactosylmonoacylglycerol; MGDG, monogalactosyldiacylglycerol; PA, phosphatidic acid; PC, phosphatidylcholine; PE, phosphatidylethanolamine.

Overall, PDEVs from edible fruits and vegetables benefit from abundant raw materials and relatively mature isolation protocols. Their long history of dietary use ensures a favorable biosafety profile, facilitating large-scale production and the development of functional foods, including functional beverages, while also conferring considerable therapeutic potential in various disease models [26, 98]. PDEVs derived from medicinal plants are frequently constrained by scarce resources and low yields, yet they often exhibit more potent bioactivity. Consequently, research in this area has predominantly focused on high-value therapeutic applications [10]. Notably, medicine-food homologous plants combine the advantages of both categories, positioning them as one of the most promising frontiers for both current and future translational research.

## Construction of drug delivery systems

### Drug type

#### Small-molecule drugs

PDEVs serve as natural drug delivery vehicles, suitable for transporting a variety of plant bioactive compounds such as luteolin [85], isoliquiritigenin [101], curcumin [54], oridonin [102] and others. PDEVs enhance the solubility and bioavailability of small-molecule drugs while simultaneously protecting them from degradation by gastric acid, thereby improving their stability. For instance, PDEVs extracted from sesame leaves were loaded with luteolin via sonication-co-incubation, demonstrating that sesame-derived PDEVs significantly enhance the aqueous solubility and dispersibility of encapsulated luteolin. These nanovesicles also maintain structural integrity under variations in thermal, photolytic, pH and ionic strength conditions, as well as simulated digestion environments [85]. PDEVs possess inherent targeting capabilities, which enhance the therapeutic efficacy of drugs. Following oral administration, GDEVs loaded with curcumin specifically target the colon region, exhibiting prolonged retention (> 48 h). These PDEVs also demonstrate significantly higher cumulative release in colonic fluid compared to free curcumin controls, thereby extending the duration of drug action and improving therapeutic outcomes [54]. Additionally, a study demonstrated that PDEVs derived from *Rabdosia rubescens* exhibit high loading efficiency (76.4 ± 3.2%) when delivering their homologous drug, oridonin [102]. This finding indicates that PDEVs are promising carriers for loading homologous drugs.

#### Proteins

Protein therapeutics often exhibit high molecular weight, structural complexity, poor stability and susceptibility to degradation, resulting in suboptimal intracellular delivery efficiency and inadequate targeting specificity [103]. PDEVs protect protein therapeutics from enzymatic degradation and environmental disruption through their bilayer membrane structure, enhancing drug stability and bioavailability. Compared with free proteins, PDEVs loaded with bovine serum albumin or heat shock protein 70 (HSP70) were taken up much more efficiently by human colon cancer cells. Furthermore, *in vivo* studies showed that bovine serum albumin-loaded PDEVs were internalized by multiple tissues, including the lung, bladder and liver [104]. In another research, PDEVs derived from grapefruit and tomato can encapsulate the HSP70 using a hybrid passive-active loading strategy, achieving targeted delivery to glioma cells. Fluorescence labeling revealed that the delivery efficiency of grapefruit-derived PDEVs significantly surpassed that of free protein, with no detectable cytotoxicity observed [105].

#### Nucleic acids

PDEVs also deliver various nucleic acids, such as dsRNA, miRNA, mRNA, siRNA and others [43]. Nucleic acid drugs carry a negative charge and are highly hydrophilic, which makes them inherently challenging to penetrate cell membranes and access the intracellular compartment. Additionally, unmodified nucleic acids are prone to enzymatic degradation and exhibit low stability [106]. By shielding RNA from degradation and mediating receptor-dependent target cell recognition, PDEVs substantially improve delivery efficiency [107]. For instance, PDEVs derived from *Brucea javanica* fruits delivered 10 endogenous functional miRNAs to triple-negative breast cancer cells, achieving 46.4% cellular uptake within 12 h, primarily through clathrin-mediated endocytosis. This led to significant suppression of both tumor growth and metastasis [108].

### Loading methods

#### Co-incubation

The co-incubation procedure is relatively straightforward, typically involving the incubation of purified PDEVs with the drug at a specific mass ratio for a defined period (usually 4–24 h) at either 37°C or 4°C. Following incubation, unbound drug is removed by centrifugation or ultrafiltration [109–111]. This passive loading method, which relies on drug diffusion and hydrophobic interactions with the PDEV lipid bilayer, is compatible with a wide range of drug types, including small-molecule compounds, proteins and nucleic acids [6]. Due to their negatively charged surface, PDEVs exhibit enhanced loading efficiency for positively charged molecules through electrostatic interactions [14]. One advantage of the co-incubation method is that it effectively preserves the integrity of the PDEVs membrane structure while also reducing preparation costs. However, its limitations include a relatively long incubation time and difficulty in controlling drug-loading stability. Notably, at lower temperatures (4°C), incubation for 1 h resulted in significantly lower drug loading compared to a 48-h incubation. Conversely, at higher temperatures (37°C/25°C), the opposite trend was observed [112]. This discrepancy may be attributed to increased temperature affecting membrane fluidity and permeability of PDEVs, with prolonged incubation at elevated temperatures potentially leading to the leakage of encapsulated drugs.

#### Extrusion

Extrusion enables efficient drug encapsulation by repeatedly forcing an aqueous solution of PDEVs and therapeutic agents through porous membranes with pore diameters ranging from 100 to 400 nm. This mechanical process induces transient disruption and reorganization of vesicles, thereby facilitating high-efficiency payload incorporation [102]. The method is also simple to operate and highly controllable, allowing for the production of drug-loaded PDEVs with uniform particle size and making it particularly suitable for small-molecule drugs. For example, using physical extrusion, Wei *et al*. [102] obtained oridonin-loaded *Rabdosia rubescens*-derived PDEVs with a size of 381.4 ± 32.5 nm, without compromising the lipid bilayer structure. However, repeated extrusion may damage membrane proteins of PDEVs, potentially compromising their targeting capability.

#### Ultrasonication

Ultrasonication typically involves mixing PDEVs with drug molecules under ultrasonic irradiation for a defined duration, followed by purification via ultrafiltration to remove unencapsulated drugs. Alternatively, cyclic sonication of the PDEV-drug mixture can be performed, with subsequent purification using ultrafiltration or size-exclusion chromatography to eliminate free drug molecules [101, 113]. For example, tomato fruit-derived PDEVs were mixed with calcitriol and subjected to six cycles of ultrasonic treatment (30 s per cycle with a 2-min interval between cycles). After completion of the cycles, the mixture was incubated at room temperature for 30 min to restore membrane integrity and remove unbound calcitriol. UV-spectrophotometry was then employed to determine the drug loading efficiency, which was measured as 47.30% ± 1.6% [113]. This method leverages ultrasound-induced cavitation, mechanical perturbation and other disruptions to the lipid bilayer to form transient pores that facilitate drug entry into the PDEV interior. It is applicable to both protein-based and small-molecule drugs. While the ultrasound method offers higher loading efficiency and faster processing speed compared to electroporation, its high-frequency vibrations may compromise the structural integrity of PDEVs, potentially affecting the activity of endogenous cargo. Studies have demonstrated that the drug-to-carrier ratio, sonication duration and ultrasonic power significantly influence drug loading capacity [114]. Therefore, when employing the ultrasonic method for drug loading, it is essential to optimize operational parameters to suit the specific requirements of different therapeutic agents.

#### Electroporation

The electroporation method involves mixing purified PDEVs with the drug in an electroporation buffer. This mixture is then subjected to pulsed electrical stimulation under specific voltage and capacitance conditions. After electroporation, the sample is incubated in a 37°C water bath for a defined period (30–60 min) to facilitate resealing of the membrane structure. Unencapsulated drug is subsequently removed via high-speed centrifugation [115, 116]. Electroporation utilizes electrical pulses to alter the permeability of the PDEV membrane, facilitating the uptake of nucleic acids and small-molecule drugs. This method is rapid and processes large quantities of PDEVs in a single procedure but requires careful parameter optimization to avoid irreversible membrane damage [117]. Despite its advantages, electroporation demonstrates limited loading efficiency for encapsulating cargo into PDEVs. For instance, the dsRNA loading efficiency in citrus-derived PDEVs was reported to be only 6.0% [115]. Similarly, the encapsulation efficiency of CX5461 into *Sophora Flavescens*-derived PDEVs using electroporation reached only 23% [118].

#### Chemical transfection

Chemical transfection is commonly employed for loading nucleic acid drugs. This typically involves mixing the transfection reagent with nucleic acids according to the manufacturer’s instructions, followed by co-incubation with PDEVs at 37°C for a defined period. Unbound nucleic acids are then used to obtain drug-loaded PDEVs [111]. Chemical transfection relies on specific transfection reagents to facilitate the delivery of therapeutic agents into delivery vehicles. The efficiency is significantly influenced by factors such as cell type, reagent selection, nucleic acid-to-reagent ratio and other experimental variables [119]. This method achieves high loading efficiency, as evidenced by an 80% drug-loading efficiency, assessed via fluorescence intensity [120]. Compared to physical methods (e.g. electroporation, ultrasonication), chemical transfection causes less structural damage to PDEVs and simplifies operational procedures. However, the potential cytotoxicity of transfection reagents may impair the biological functions of PDEVs, and residual reagents could compromise the safety profile of drug delivery systems.

#### Freeze-thaw cycles

**Figure 2:**
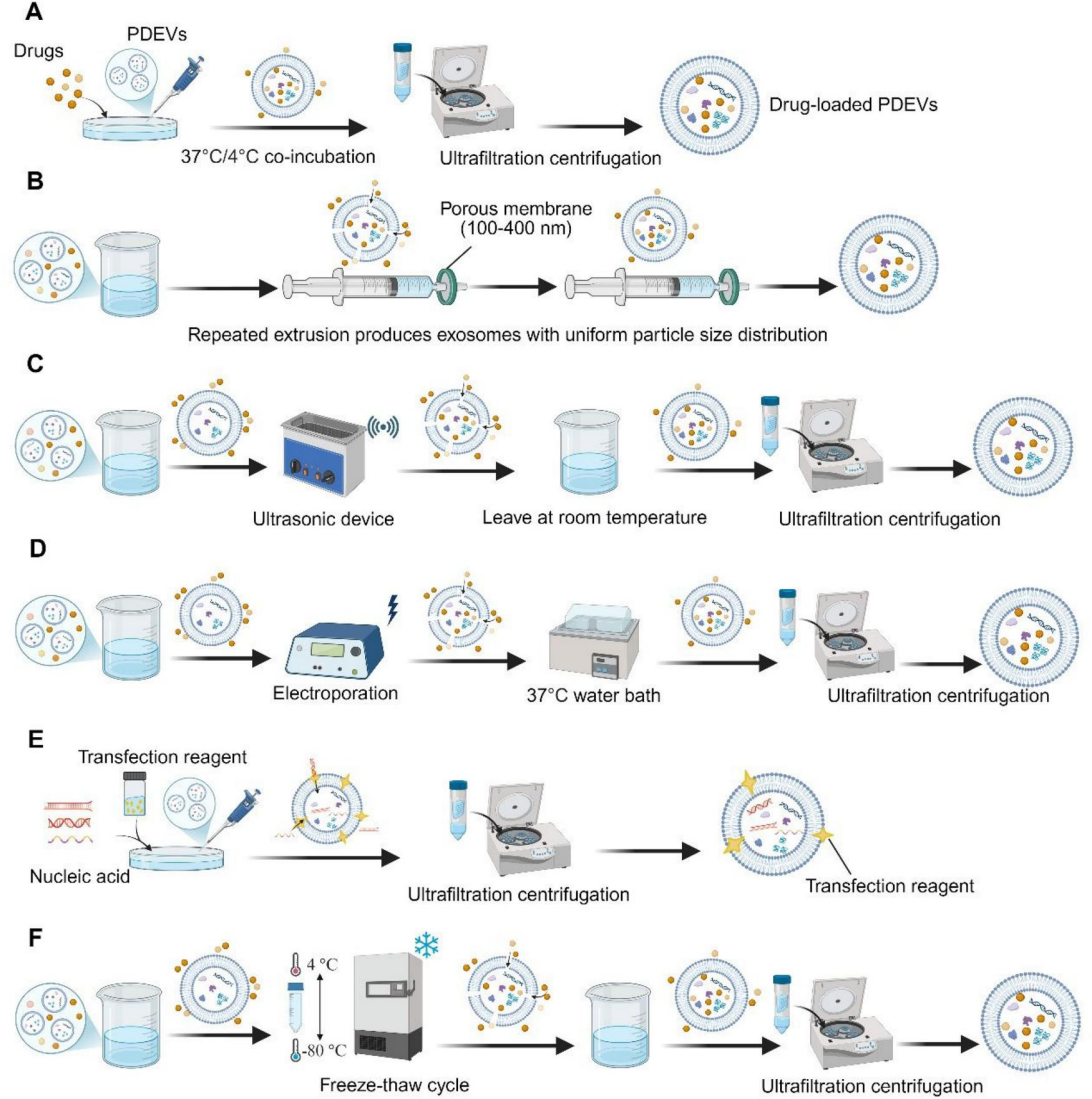
Drug loading methods for plant-derived extracellular vesicles. (**A**) Co-incubation; (**B**) extrusion; (**C**) ultrasonication; (**D**) electroporation; (**E**) chemical transfection; (**F**) freeze-thaw cycles. Created with BioRender.com.

The freeze-thaw method utilizes rapid freezing to induce ice crystal formation within PDEVs, thereby disrupting their surface membranes. During subsequent thawing, the damaged membranes self-seal through lipid reformation. Repeated freeze-thaw cycles between –80°C and room temperature provide multiple opportunities for target molecules to enter the vesicular interior, ultimately enhancing encapsulation efficiency [121]. For example, when subjected to three freeze-thaw cycles alternating between –80°C and 4°C, tomato-derived PDEVs achieved a calcitriol encapsulation efficiency of 34.8% [113]. The freeze-thaw method is particularly suitable for thermolabile drugs due to its relatively mild processing conditions, which avoid the use of chemical reagents and intense physical treatments, thereby effectively preserving drug activity. However, repeated freeze-thaw cycles may inflict cumulative damage on PDEVs and often induce vesicle aggregation. These effects can potentially compromise encapsulation efficiency and cause spatial heterogeneity in drug distribution within vesicles [92]. The drug loading process is illustrated in Figure 2.

#### Others

Beyond conventional methods, researchers are exploring novel PDEV-based drug delivery strategies, including osmotic shock-based loading, magnetic stirring-assisted encapsulation, light-irradiation-mediated drug loading and microfluidic device-enabled drug embedding (Table 3). The osmotic shock approach leverages cationic interactions and controlled osmotic stress, making it particularly suitable for oral mRNA vaccine delivery [122]. For example, loading SARS-CoV-2 mRNA into orange (*Citrus sinensis*) juice-derived PDEVs via osmotic shock achieved a drug-loading efficiency of 72 ± 11% [123]. Magnetic stirring-assisted encapsulation increases drug loading efficiency with prolonged incubation time. For instance, the drug loading efficiency of curcumin into GDEVs reached 36.8% after 1.5 h of incubation [124]. Microfluidic devices allow precise control over the particle size of siRNA-PDEV complexes; however, siRNA aggregation remains a challenge, leading to relatively low drug loading efficiency. For instance, grapefruit-derived PDEVs exhibited only 11% siRNA loading efficiency under microfluidic processing conditions [125]. Light-triggered drug loading is an innovative technique that utilizes the photosensitive properties of PDEVs. Upon light irradiation, these vesicles generate reactive oxygen species (ROS), which transiently enhance membrane permeability, thereby enabling controlled drug encapsulation. For instance, *Pueraria lobata* leaf-derived PDEVs possess inherent photosensitizing properties. Under optimized vesicle-to-drug ratio conditions (1:30), irradiation with specific-wavelength light for 10 min achieves an encapsulation efficiency of up to 80% for fluorescein isothiocyanate-labeled dextran (70 kDa), while maintaining structural integrity and stability for over 30 days [126].

**Table 3:** Advantages and disadvantages of different drug loading methods.

Methods	Mechanism	Key factors	Advantages	Disadvantages	Applications	References
Co-incubation	Passive diffusion	Drug concentration, incubation temperature and time, pH	Simple operation, no specialized equipment needed Preserves vesicle structural integrity	Time-consuming	Small-molecule drugs Proteins Nucleic acids	[14]
Extrusion	Mechanical shear-induced membrane deformation	Membrane pore size, extrusion cycles	Simple operation Enables control over uniform vesicle size	Shear forces may damage membrane proteins Multiple extrusions may cause drug leakage, affecting loading efficiency	Small-molecule drugs	[102]
Ultrasonication	Ultrasound-induced cavitation	Sonication power, frequency, drug/carrier ratio, sonication time	High drug loading efficiency Rapid operation	May damage plant vesicle structure High-frequency vibration may cause vesicle aggregation	Proteins Hydrophilic small-molecule compounds	[114]
Electroporation	Electric pulse-induced membrane poration	Electric field strength, buffer, pulse duration	Simple and rapid operation No chemical reagents required	Requires specialized equipment (electroporator) Low loading efficiency Requires parameter control to prevent cell membrane damage	Nucleic acids Small-molecule drugs	[117]
Chemical transfection	Electrostatic interaction	Transfection reagent, nitrogen-to-phosphorus ratio, incubation time	Simple operation Relatively high loading efficiency	Transfection reagents are toxic May affect drug bioactivity and loading stability	Nucleic acids	[119]
Freeze-thaw cycles	Freeze-thaw cycles induce transient membrane pore formation	Freeze-thaw cycles, cryoprotectant	Simple operation with good reproducibility Relatively high loading efficiency	May cause membrane damage and vesicle aggregation	Small-molecule compounds (e.g., thermosensitive drugs)	[92]
Osmotic shock-based loading	Cationic interactions and controlled osmotic stress	Osmotic pressure gradient, cation type and concentration	High loading efficiency Preserves vesicle structural integrity	Cationic materials alter zeta potential of plant-derived extracellular vesicles, potentially affecting circulatory stability *in vivo*	Nucleic acids	[123]
Magnetic stirring	Passive diffusion	Stirring speed and time, drug concentration	Simple operation Maintains vesicle structural integrity	Time-consuming	Small-molecule drugs	[124]
Microfluidic technology	Determined by microfluidic chip design (e.g., shear-induced membrane permeabilization, electroporation)	Chip geometry, flow parameters (e.g., flow rate ratio)	Precise size control Scalable manufacturing	High equipment dependency Low loading efficiency Loss of membrane proteins impairing drug targeting	Nucleic acids	[125]
Light-triggered drug loading	Photosensitizer-mediated reactive oxygen species generation induces transient membrane permeabilization	Irradiation time, wavelength, drug-to-carrier ratio	High loading efficiency Maintains vesicle integrity Excellent long-term stability of drug-loaded system	Risk of photodamage to drugs Prolonged irradiation (>15 min) disrupts membrane structure	Small-molecule drugs Proteins	[126]

### Endogenous and exogenous loading

Endogenous drug loading refers to the process of incorporating therapeutic agents into PDEVs during their natural secretion. This can be achieved through two principal strategies: engineering the donor plant’s genome to drive *de novo* synthesis; manipulating the physiological microenvironment (e.g. osmotic pressure) to promote active uptake and packaging. Urbanelli *et al*. [127] genetically modified tobacco to express the neomycin phosphotransferase II gene and isolated PDEVs from the apoplastic fluid. These vesicles were found to carry transgenic mRNA rather than DNA, demonstrating that PDEVs protect transgenic mRNA from degradation and that genetic engineering can achieve endogenous encapsulation of exogenous nucleic acids. Shkryl *et al*. [128] further revealed that PDEVs can deliver artificial miRNAs into recipient cells to induce post-transcriptional gene silencing. Collectively, these findings validate the feasibility of engineering transgenic plants for the production of therapeutic proteins and regulatory small RNAs, paving the way for developing innovative oral RNA nanotherapeutics. Nevertheless, two key concerns remain. First, RNA sorting into PDEVs is regulated in a sequence-selective manner and constrained by competitive loading effects [127, 128]. Second, the environmental dissemination of PDEVs harboring antibiotic resistance genes poses potential ecological safety risks [127]. Another study [129] reported that hypotonic induction using indocyanine green (ICG) stimulated aloe cells to secrete more PDEVs as a defense mechanism against cellular stress. These PDEVs were confirmed to be successfully loaded with ICG, exhibiting color and photothermal properties identical to those of free ICG solution. Lipidomic and proteomic analyses revealed that PDEVs contained more long-chain lipids (including phospholipids and sterol esters) and fewer intravesicular proteins, resulting in enhanced structural integrity.

Exogenous loading refers to the process of incorporating therapeutic agents into PDEVs after their isolation and purification. Exogenous loading has been studied more extensively than the endogenous approach, presumably because it offers a wider array of loading techniques, such as co-incubation, ultrasonication and electroporation, which contribute to its broader applicability. However, the selection of loading modality exerts a decisive influence on both the entrapment efficiency and the final drug payload, as summarized in Table 4. Notably, endogenous loading confers superior drug-loading efficiency and vesicular stability compared with exogenous modalities. For example, using the endogenous loading protocol with ICG-hypoosmotic stress, aloe-derived PDEVs afforded an ICG payload of 223.4 μg mL^−1^, marking a 2.8-fold increase over exogenous methods such as ultrasonication or co-incubation. Simultaneously, PDEVs obtained by hypoosmotic stress exhibited increased rigidity and an elevated phase-transition temperature, enabling deeper and more extensive penetration in 3D tumor spheroids and *in vivo* models, and thereby producing markedly enhanced photothermal therapeutic efficacy [129]. This suggests that endogenous loading is a more promising strategy for drug encapsulation.

**Table 4:** Compatibility of drug types with loading strategies.

Plant source	Loaded drug	Drug type	Loading method	Loading capacity, %	Loading efficiency, %	References
*Catharanthus roseus* flower	Vincristine	Small-molecule	Co-incubation	12.4	84.6	[56]
Celery (*Apium graveolens* L.)	Doxorubicin	Small-molecule	Co-incubation	NA	87.03	[109]
Garlic	Ascorbic acid	Small-molecule	Co-incubation	13.5 ± 0.4	78.1 ± 2.8	[110]
Ginger				14.8 ± 0.2	87.2 ± 1.4	
Turmeric				14.2 ± 0.3	82.5 ± 2.1	
*Rabdosia rubescens*	Oridonin	Small-molecule	Physical extrusion	9.21 ± 0.45	76.4 ± 3.2	[102]
Broccoli	Astaxanthin	Small-molecule	Ultrasonication	6.824	NA	[114]
Sesame leaves	Luteolin	Small-molecule	Ultrasonication	20.5	∼ 91.9	[85]
*Lycium barbarum* L.	Isoliquiritigenin	Small-molecule	Ultrasonication	13.5 ± 0.27	82.6 ± 4.19	[101]
Ginger	Curcumin	Small-molecule	Ultrasonic incubation	94.027 ± 0.094	89.300±0.344	[54]
Grapefruit (*Citrus* × *paradisi*) and tomato (*Solanum lycopersicum*) juices	Heat shock protein 70	Protein	Ultrasonication, co-incubation	NA	1.10	[105]
Cabbage (*Brassica oleracea* var. *capitata* L. and *Brassica oleracea* var. *capitata* F. *rubra*)	Doxorubicin, miRNA	Small-molecule drug, nucleic acid	Co-incubation, chemical transfection	NA	NA	[111]
Citrus fruits (lane late navel orange)	dsRNA	Nucleic acid	Electroporation	NA	6.0	[115]
*Sophora Flavescens*	CX5461	Small-molecule	Electroporation	NA	23	[118]
Ginger	siRNA	Nucleic acid	Chemical transfection	NA	80	[120]
Tomato fruit	Calcitriol	Small-molecule drug	Passive loading	NA	15.4	[113]
			Freeze-thaw cycles		34.8	
			sonication		47.3	
Orange (*Citrus sinensis*) juice	SARS-CoV-2 mRNA	Nucleic acid	Osmotic shock-based loading	NA	72 ± 11	[123]
Ginger	Curcumin	Small-molecule drug	Magnetic stirring	NA	36.8	[124]
Grapefruit	siRNA	Nucleic acid	Microfluidic device	NA	11	[125]
*Pueraria lobata* leaf	Fluorescein isothiocyanate-dextran	Fluorescence-labeled small molecular cargo	Photoirradiation	NA	80	[126]

### Surface modification

**Figure 3:**
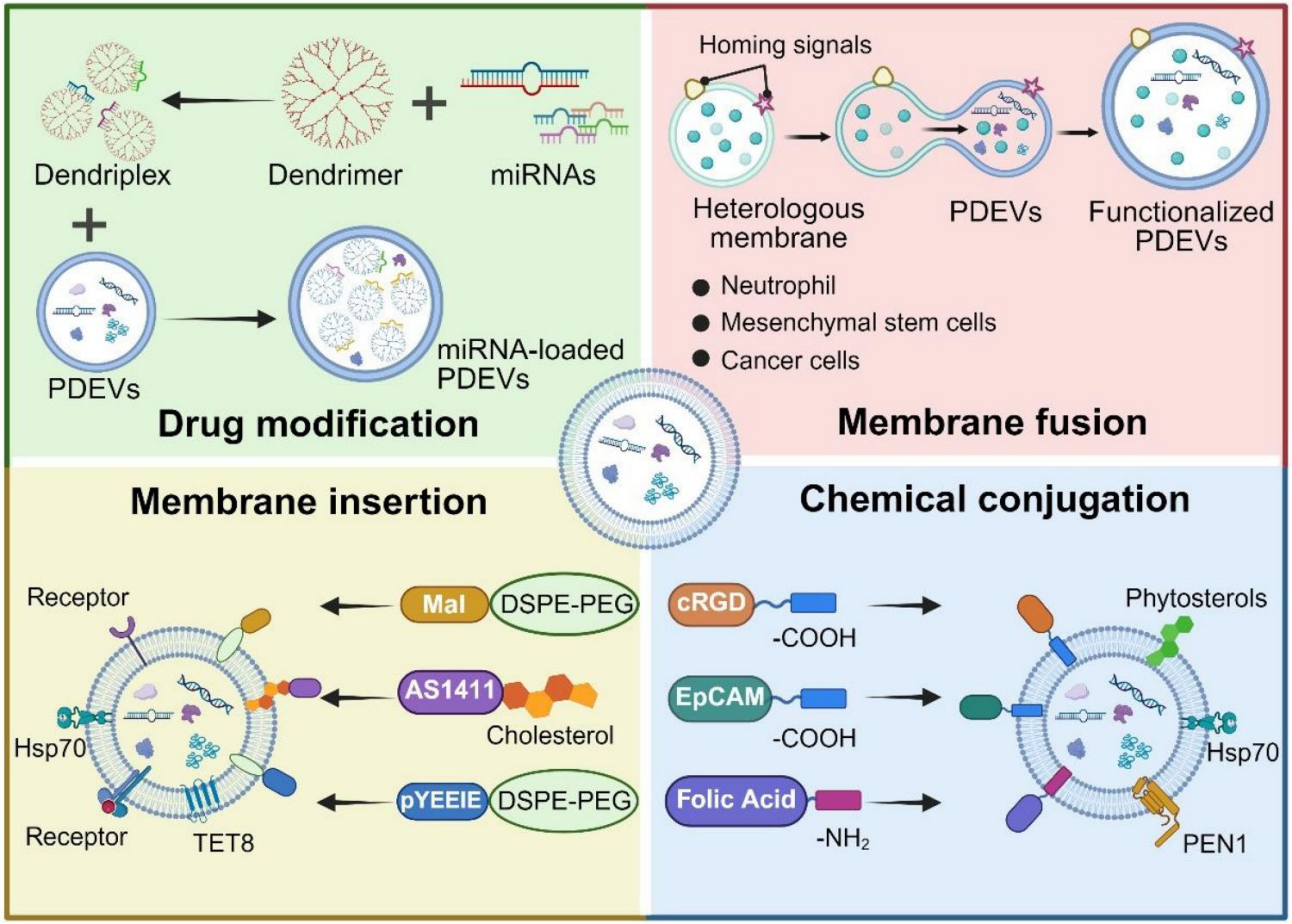
Surface modification of plant-derived extracellular vesicle-based drug delivery system.

To enhance the drug loading efficiency and targeting capability of PDEV-based drug delivery systems (including PDEVs and drug molecules), surface modification of the system is commonly employed (Figure 3). Surface-modified drug molecules exhibit enhanced encapsulation efficiency in PDEV-based delivery systems. This is particularly critical for nucleic acid therapeutics, which otherwise suffer from electrostatic repulsion against the negatively charged PDEV membrane. For instance, the complex of miR-146a and polyamidoamine dendrimers achieved a loading efficiency of 96–98% in watermelon-derived PDEVs [130]. In another study, miR-17 complexed with polyethylenimine (PEI) achieved an encapsulation efficiency of 86% in grapefruit-derived PDEVs, compared with only 5.9% without PEI [131]. Furthermore, *in vivo* experiments revealed that combining PEI/RNA complexes with grapefruit-derived PDEVs prevented the brain inflammation typically induced by PEI. These findings demonstrate the effectiveness of cationic polymers in loading nucleic acid drugs and highlight the potential of PDEVs in mitigating the toxic effects of synthetic carriers.

Optimizing the plant-derived extracellular vesicle (PDEV)-based delivery system involves engineering both its components. Drug modification improves loading efficiency; surface functionalization of the PDEV, employing methods like membrane fusion, lipid insertion or chemical conjugation, enables active targeting. Created with BioRender.com.

Compared to the direct modification of drugs, the surface engineering of PDEVs has been more extensively explored. Methods such as ligand conjugation and membrane fusion enable the surface modification of PDEVs with biomolecules (e.g. targeting peptides, folic acid), thereby enhancing their active targeting capability, circulatory stability and cellular uptake efficiency. Ligand conjugation to PDEVs is primarily achieved through chemical conjugation and membrane insertion. Native carboxyl and amino functional groups on PDEV surfaces provide ready-made conjugation handles for direct chemical ligation. For instance, the epithelial cell adhesion molecule (EpCAM) aptamer was covalently conjugated to carboxyl groups on *Cucurbita moschata*-derived PDEVs using standard carbodiimide chemistry, demonstrating these inherent handles for targeted functionalization of biomimetic nanocarriers [132]. In membrane insertion, functionalized lipids bearing reactive headgroups are incorporated hydrophobically into the membrane bilayer to display specific ligands on the PDEV surface [47, 82, 133]. For example, the lipophilic nature of 1,2-distearoyl-sn-glycero-3-phosphoethanolamine (DSPE) was exploited to anchor a DSPE-Polyethylene glycol (PEG) 2000-pYEEIE conjugate into Rhodiola-derived PDEVs, enabling stable display of the targeting peptide on the vesicle surface [133]. Similarly, hydrophobic insertion was used to anchor the maleimide-functionalized lipid DSPE-PEG2000-Mal onto grapefruit-derived PDEV membranes. A 3′-thiol-modified aptamer (e.g. R11-3) was subsequently conjugated to the terminal maleimide via a thiol-maleimide click reaction, yielding a nanovehicle that targets human brain microvascular endothelial cells [82]. Beyond DSPE, cholesterol serves as another commonly used membrane-anchoring molecule. Researchers constructed an arrowhead RNA three-way junction conjugated with folate and cholesterol at its opposite termini. Leveraging the hydrophobic property of cholesterol, this engineered complex was incorporated into GDEV membranes, thereby enabling the directional presentation of the folate ligand on the vesicular surface [120]. Membrane fusion is currently utilized to construct engineered PDEVs. For instance, ginseng root-derived PDEVs fused with neutrophil membranes acquire the innate neutrophil tropism for inflammatory lesions [134]; likewise, grapefruit-derived PDEVs fused with nanovesicles from C-C motif chemokine receptor 6 (CCR6)-overexpressing gingival mesenchymal stem cells facilitate targeted drug delivery via the CCR6/CCL20 chemotactic axis [135]. Additionally, the fusion with breast-cancer-cell membrane fragments endows lemon-derived PDEVs with homologous targeting, thereby increasing cellular uptake and robustly suppressing 4T1 tumor growth [136].

### Composite drug delivery system

The integration of PDEVs with hydrogels creates a synergistic bioactive delivery system, where hydrogels protect the structural and functional integrity of PDEVs and enable controlled release. Meanwhile, the bioactive molecules and therapeutic agents carried by PDEVs actively modulate the local microenvironment, playing pivotal roles in tissue repair and immunomodulation. For example, polydopamine-modified hydrogels encapsulating *Flos Sophorae Immaturus*-derived PDEVs (rich in rutin) can be locally implanted at spinal cord injury sites. These hydrogels modulate the oxidative stress microenvironment, resulting in synergistic effects that significantly promote motor function recovery, improve voiding dysfunction and accelerate neural regeneration [90]. Oridonin-loaded PDEVs integrated with semi-interpenetrating polymer network hydrogels for treating chemoradiotherapy-induced stomatitis extend local drug retention for up to 7 h, while also demonstrating favorable biocompatibility (hemolysis ratio < 2%) and antimicrobial activity [102]. Additionally, the integration of environmentally responsive hydrogels with PDEVs enhances their targeting precision and therapeutic efficacy within pathological microenvironments. Tan *et al*. [137] developed a biodegradable, wireless thermoelectric hydrogel (HFN) co-crosslinked from hyaluronic acid and Pluronic F127, which encapsulates REDV peptide-modified ginseng-derived exosomes (RGE). This HFN-RGE composite delivery system reconstructs a biomimetic endogenous electric field through a ‘cation trap’ effect, effectively promoting angiogenesis and tissue regeneration in diabetic ulcers.

**Figure 4:**
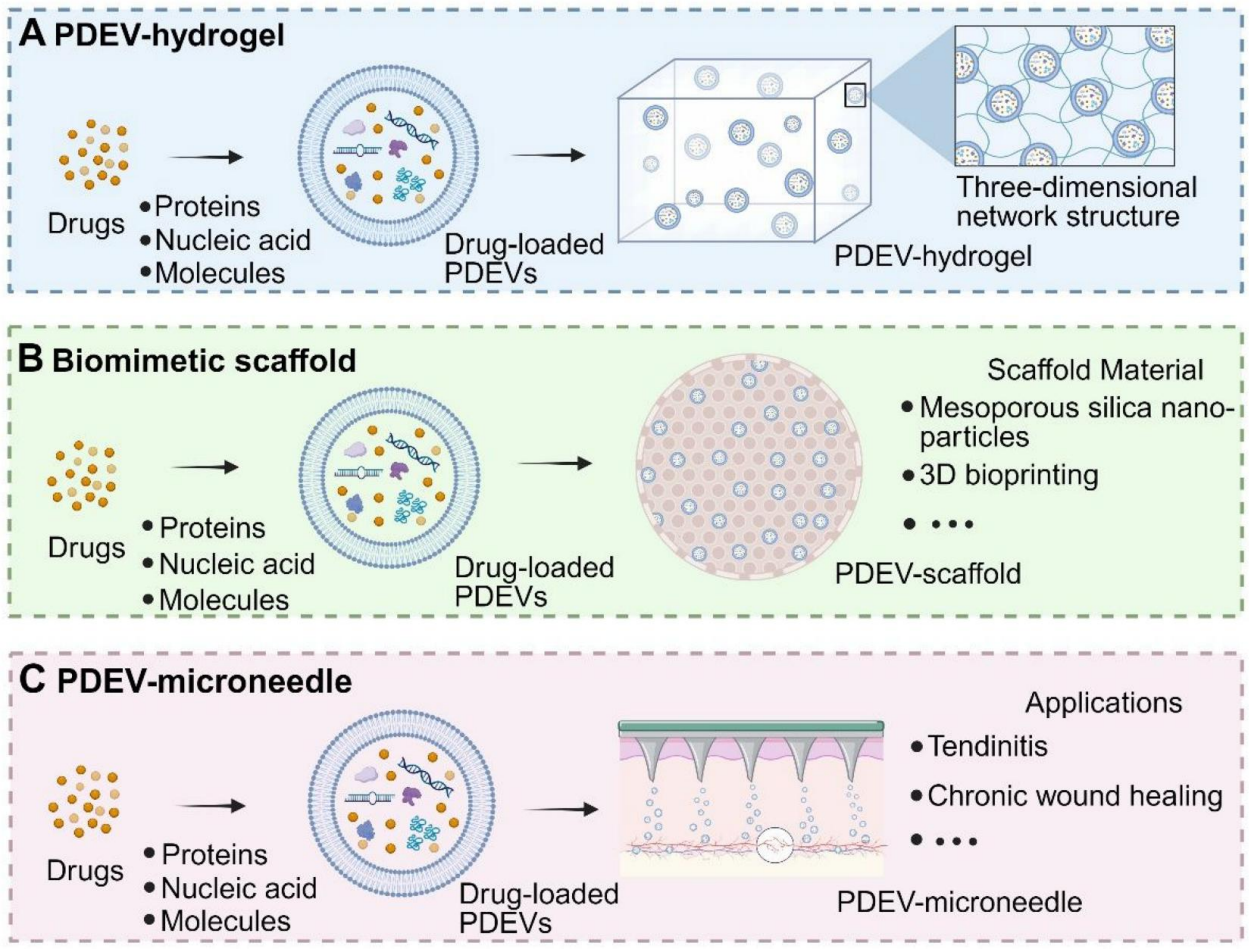
Composite drug delivery system. (**A**) Encapsulation of plant-derived extracellular vesicles (PDEVs) in hydrogels; (**B**) Encapsulation of PDEVs in scaffolds; (**C**) Combination of PDEVs and microneedles. Created with BioRender.com.

Recently, bioinspired scaffolds integrating PDEVs with other materials have garnered increasing attention for disease therapy (Figure 4). In the treatment of inflammatory bowel disease, a composite scaffold constructed from GDEVs-coated large mesoporous silica nanoparticles not only significantly enhances the oral bioavailability of the antibody infliximab by stabilizing its conformation, resisting gastrointestinal degradation and enabling colon-targeted delivery, but also synergistically inhibits TNF-α release. By leveraging GDEVs’ intrinsic activity to block the inflammasome, the therapy surpassed the efficacy of intravenous injection in mouse models [138]. The integration of isoliquiritigenin (ISL)-loaded PDEVs from *Lycium barbarum* L. into a 3D-printed gelatin methacryloyl hydrogel scaffold for spinal cord injury repair not only prolongs the sustained release of ISL but also synergistically combines PDEVs’ inherent anti-inflammatory properties with ISL to promote neural stem cell differentiation and synaptic growth. This approach also induces microglial polarization from the pro-inflammatory M1 to the reparative M2 phenotype, culminating in significantly restored motor function and reduced glial scar formation in rats [101]. These findings indicate the tremendous potential of the PDEV-scaffold composite system in drug delivery for immunomodulation and tissue repair.

Additionally, the combination of PDEVs with microneedles has demonstrated promising therapeutic potential in the treatment of tendinitis [139] and chronic diabetic wounds [140]. This proven success paves the way for the development of composite drug-delivery systems that integrate PDEVs with other materials—such as electrospun fibers, liposomes and sponges—to broaden their clinical translation and applications.

### Evaluation of drug delivery systems

The evaluation of drug delivery systems generally encompasses physicochemical characterization (e.g. morphology, particle size, zeta potential and stability), drug loading and release performance (e.g. loading capacity, loading efficiency and release behavior), biological assessment (e.g. efficacy, pharmacokinetics and toxicity), as well as clinical translation potential (e.g. preparation process, cost and storage stability).

The morphology of PDEV drug delivery systems is typically characterized by atomic force microscopy (AFM), transmission electron microscopy (TEM) and scanning electron microscopy (SEM), with their size distribution and zeta potential measured by nanoparticle tracking analysis (NTA) and dynamic light scattering (DLS) [34, 48, 105, 114, 141–143]. SEM enables assessment of morphology and size distribution of PDEVs, while TEM complements this by allowing direct visualization of fine morphology and bounding membrane integrity [34, 53, 105, 141]. AFM further provides nanoscale three-dimensional topographic imaging, from which particle height and surface roughness can be quantified, providing complementary information on vesicle topography [105, 143]. Following drug loading, PDEV morphology remains largely intact, accompanied by an increase in particle size and a shift in zeta potential. For instance, after loading CX5461, the particle size of PDEVs derived from *Sophora Flavescens* increased from 80 nm to 110 nm, and the zeta potential changed from –32 mV to –20 mV [118]. Similarly, the particle size of avocado-derived PDEVs loaded with doxorubicin increased from 99.58 ± 5.09 nm to 151.2 ± 6.36 nm, accompanied by a decrease in zeta potential from –17 mV to –28 mV [144]. Thus, alterations in particle size and zeta potential can serve as indirect indicators of successful drug loading.

Drug loading in PDEVs is typically quantified by loading capacity (LC; also termed drug loading content) and encapsulation efficiency (EE; also termed loading efficiency). LC is defined as the mass ratio of entrapped drug to the total carrier system, whereas EE denotes the percentage of the initial drug dose that is successfully entrapped. Both calculations depend on accurate drug quantification, routinely accomplished by ultraviolet-visible (UV) spectrophotometry [94, 145], high-performance liquid chromatography (HPLC) [101, 102, 113, 144] and microplate readers [146] operating via absorbance, chromatography or fluorescence readouts. UV spectrophotometry offers simplicity and low cost for pure, UV-absorbing drugs but lacks selectivity due to spectral overlap. HPLC enables multi-component analysis of complex samples, albeit at higher cost and longer run times. Microplate readers offer high throughput and low sample consumption for batch analysis; however, without prior separation, they only provide total signals when using conventional absorbance or fluorescence detection. Drug release is typically assessed by dynamic dialysis [101, 113], stirring [144], diffusion cells [112] or Transwell inserts [137, 147]. Dynamic dialysis and the stirring method are most commonly used to evaluate the *in vitro* drug release behavior of conventional formulations owing to their operational simplicity. The release medium is typically PBS, and PBS of different pH values can be used to evaluate the pH‑dependent release behavior of PDEVs [148, 149]. The diffusion cell method is specifically designed to evaluate drug permeation across the skin barrier [112]. Transwell inserts are commonly used to evaluate the sustained release and structural protection of hydrogels for PDEVs [137, 147]. By physically separating the hydrogel from the receiving medium, they enable dynamic quantification of released PDEVs and facilitate subsequent assessment of vesicle integrity.

Despite considerable progress in biological evaluation focusing on efficacy and mechanisms, the clinical translation of PDEV drug carriers has lagged behind. PDEVs are efficiently internalized by diverse animal cells and tissues—such as intestinal epithelial cells [9, 32], glial cells [150], keratinocytes [151], lungs [152] and liver [74]—though their uptake exhibits regional variations. Man *et al*. [78] employed *in situ* intestinal perfusion coupled with HPLC analysis to investigate GDEV absorption kinetics in rats, demonstrating uptake across the small intestine at concentrations spanning 15–60 mg mL^−1^ with a duodenal > jejunal > ileal efficiency gradient. However, the metabolic pathways and clearance mechanisms of PDEVs *in vivo* are still poorly understood.

## Main applications

### Tumor-targeted therapy

Chemotherapy, a widely used antitumor treatment modality, faces challenges such as cytotoxicity and insufficient therapeutic targeting of agents like doxorubicin (DOX) and paclitaxel, which may induce severe systemic adverse effects [153]. For instance, DOX can cause multi-organ toxicity, particularly cardiotoxicity [154], while paclitaxel may lead to myelosuppression with peripheral neuropathy [155]. Prolonged administration of both agents often results in drug resistance.

**Figure 5:**
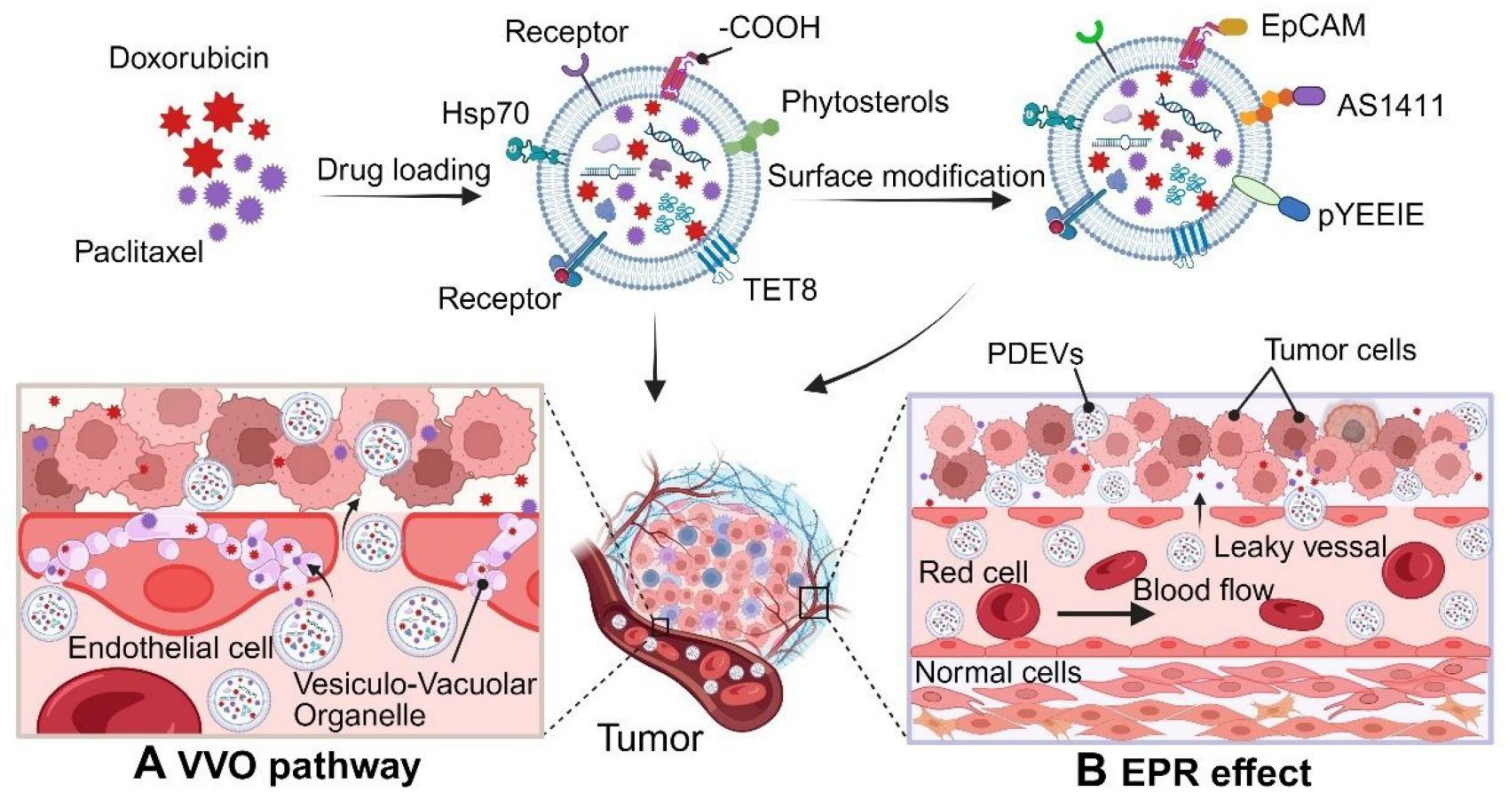
Passive accumulation mechanisms of plant-derived extracellular vesicles in tumor tissue. (**A**) Vesiculo-vacuolar organelle pathway: owing to their nanoscale size, plant-derived extracellular vesicles (PDEVs) could efficiently target tumor tissues by exploiting the abundant vesiculo-vacuolar organelles, a transendothelial pathway predominantly activated in tumor vasculature. (**B**) Enhanced permeability and retention effect: PDEVs extravasate through the discontinuous endothelium of tumor vasculature and accumulate within the tumor interstitium, leading to passive targeting and enhanced therapeutic efficacy. Created with BioRender.com.

Due to their nanoscale dimensions, PDEVs exhibit passive tumor targeting and preferentially accumulate in tumor tissues through the enhanced permeability and retention effect and the vesicular-vacuolar pathway [156]. The process is illustrated in Figure 5. Moreover, their endogenous bioactive components can synergize with chemotherapeutic agents to enhance antitumor efficacy. The combination of *Persea americana*-derived PDEVs and DOX acts synergistically to potentiate cytotoxicity over monotherapies, as these vesicles carry endogenous anticancer proteins—such as defensins and metabolic enzymes—that inherently prime tumor cells for chemotherapeutic insult. However, this synergistic anticancer effect is cell type-dependent: its magnitude and underlying mechanism vary across cancer lineages, yielding distinct modulatory patterns within the TP53 and STAT signaling axes [144]. In MCF-7 cells, *Persea americana*–derived PDEVs alone upregulated STAT expression by approximately 480-fold while suppressing TP53; co-treatment with DOX sustained high STAT levels with partial TP53 restoration. Conversely, in 4T1 cells, both treatments suppressed TP53 and STAT [144]. These opposing, lineage-specific responses underscore the need for context-dependent evaluation in PDEV-based cancer therapy. Additionally, PDEVs can mitigate the toxic effects of chemotherapeutic agents. For instance, those derived from *Momordica charantia* L. reduce oxidative stress, preserve mitochondrial integrity and attenuate DOX-induced cardiomyocyte apoptosis through the p62/Keap1/Nrf2 axis [157]. Another study revealed that DOX loaded into *Citrus limon*-derived PDEVs exhibited comparable cytotoxicity against HeLa cells (cervical cancer cells) but significantly reduced inhibition of HEK293T (human embryonic kidney cells) compared to free DOX [146]. These findings demonstrate that PDEVs, as delivery vehicles for chemotherapeutic drugs, can effectively address limitations such as drug cytotoxicity and instability. Furthermore, they facilitate enhanced drug uptake by tumor cells, thereby providing dual benefits: improved therapeutic efficacy and significantly reduced damage to normal tissues.

However, the efficiency of this passive targeting is often compromised by tumor heterogeneity and the complex stromal barrier, resulting in uneven penetration and limited distribution of PDEVs within tumor tissue [158]. Consequently, researchers have engineered PDEVs via surface modification with targeting ligands to achieve active tumor targeting. Through a genetic membrane-anchoring strategy, the tumor-targeting peptide iRGD was stably displayed on GDEVs, significantly enhancing their cellular uptake *in vitro* and tumor accumulation *in vivo* relative to unmodified counterparts [47]. Transcriptomic analysis further revealed that these engineered PDEVs promoted cancer cell apoptosis via modulation of the cell cycle and p53 signaling pathways. Thus, drug loading into such modified PDEVs is expected to improve both the safety and stability of therapeutic agents. Surface-engineering *Rhodiola rosea*-derived PDEVs loaded with DOX using DSPE-PEG2000-pYEEIE not only enhanced DOX uptake by melanoma A375 cells and inhibited cancer cell proliferation, but also demonstrated favorable biocompatibility and a sustained release profile, with 60% cumulative release over 24 h [133]. In another study, DOX and zeolite imidazolate framework-8 were encapsulated within *Cucurbita moschata*-derived PDEVs and further functionalized with EpCAM aptamers to construct a biomimetic drug delivery system. This system specifically binds to the EpCAM receptor, which is overexpressed on prostate cancer cells, enhancing DOX uptake and accumulation in tumor cells while effectively evading phagocytic clearance, thereby improving drug delivery stability [132].

### Inflammatory diseases

Multiple components within PDEVs (e.g. miRNAs, proteins) possess inherent anti-inflammatory activity and have demonstrated favorable therapeutic efficacy in diseases such as inflammatory bowel disease [159], periodontitis [160] and pneumonia [134]. Broccoli-derived nanoparticles loaded with sulforaphane target colonic dendritic cells and induce their conversion to a tolerogenic phenotype through activation of the adenosine monophosphate-activated protein kinase signaling pathway, thereby alleviating dextran sulfate sodium-induced colitis [161]. Engineered PDEVs derived from *Panax ginseng* roots loaded with miRNA-182-5p ameliorate acute lung injury in sepsis by targeting the NOX4/Drp-1/NLRP3 signaling pathway [134].

Additionally, PDEVs can be loaded with anti-inflammatory drugs to achieve synergistic therapeutic effects. Grapefruit-derived nanovesicles (GDNs) serve as a novel oral drug delivery system for the targeted delivery of the anti-inflammatory drug methotrexate (MTX) to intestinal macrophages. These nanovesicles are internalized by macrophages via macropinocytosis and clathrin-mediated endocytosis, thereby alleviating dextran sulfate sodium-induced colitis in mice [95]. Compared to free MTX, GDNs significantly enhanced the anti-inflammatory efficacy of MTX, potentially due to synergistic interactions with inherent anti-inflammatory components within GDNs, such as phosphatidylethanolamine, phosphatidylcholines and naringin.

### Neurological diseases

The treatment of neurological disorders, such as Parkinson’s disease (PD), Alzheimer’s disease and cerebrovascular diseases, faces significant challenges due to the presence of the blood-brain barrier (BBB) [162–164]. With a demonstrated ability to cross the BBB, PDEVs naturally encapsulate multiple bioactive components, making them a highly promising class of novel drug delivery systems for neurological disorders. For instance, *Momordica charantia*-derived PDEVs contain miRNAs (e.g. miR-5266 and miR-5813) that suppress neuroinflammation and inhibit glioma progression, whereas ginseng-derived PDEVs contain bioactive lipids such as ceramides and phosphatidic acids that alleviate neuroinflammation by promoting microglial polarization toward a neuroprotective phenotype [165]. However, unlike liposomes and ADEVs, PDEVs exhibit a species-dependent capacity to traverse the BBB. Conventional liposomes generally require surface modification to achieve efficient penetration [166]. ADEVs universally cross the BBB, yet their transport rates and underlying mechanisms are highly heterogeneous [167]. Studies have shown that vesicles derived from ginger and aloe are able to permeate the BBB, and this permeability is enhanced upon loading with itraconazole, whereas vesicles derived from black cumin seeds fail to cross the barrier irrespective of drug loading [168].

Furthermore, PDEVs can modulate brain inflammation and associated disorders through the gut-brain axis. GDENs loaded with antimicrobial peptide-functionalized tetrahedral framework nucleic acids significantly ameliorate motor dysfunction and pathological manifestations in PD mice. This is achieved by specifically eliminating PD-associated pathogenic bacteria, reducing pro-inflammatory cytokine release, suppressing neuroinflammation and aberrant α-synuclein deposition, while simultaneously normalizing gut microbiota balance [169]. Targeting the microbiota-gut-brain axis represents a pivotal therapeutic strategy for neurological disorders, with PDEV drug delivery systems demonstrating significant potential. Future engineering strategies to enhance their targeting specificity, payload capacity and bioavailability will advance personalized precision treatments for neurological diseases.

### Skin-related disorders

The nanoscale properties and intrinsic lipophilicity of PDEVs enable them to penetrate the skin barrier, making them promising vehicles for transdermal drug delivery. Cucumber-derived PDEVs encapsulating lipophilic drugs (e.g. the fluorescent dye DiI) demonstrated significant transdermal efficiency in an ex vivo porcine ear skin model. The average penetration depth and the amount of drug penetrated were approximately 2-fold greater than those in the free DiI group, and they were able to access the dermis within 6 h [170]. In another study, the antifungal drug terbinafine (TBF) was encapsulated in cucumber-derived PDEVs and dispersed in Carbopol 940 gel (TBF-PEV@Gel) for treating cutaneous fungal infections. Experimental results demonstrated that, within 24 h, the TBF-PEV group achieved a cumulative transdermal permeation of 14.34 μg cm^−2^, 4.57-fold higher than that of the free TBF group, whereas the TBF-PEV@Gel group exhibited 9.73 μg cm^−2^ [112]. This indicates that TBF-PEV exhibits excellent transdermal permeability, while the Carbopol gel moderately retards drug diffusion. Further analysis revealed that cucumber-derived PDEVs enhance transdermal delivery efficiency by disrupting the ordered lipid arrangement of the stratum corneum and the spatial conformation of keratin, unlike free drugs, which rely primarily on passive diffusion, thereby facilitating drug penetration through both transcellular and intercellular pathways into deep skin layers [112]. Moreover, a case series suggests that rose stem cell-derived PDEVs are effective in alleviating atopic dermatitis, accelerating wound healing and scar remodeling, reducing hyperpigmentation and enhancing recovery following laser procedures [171].

**Figure 6:**
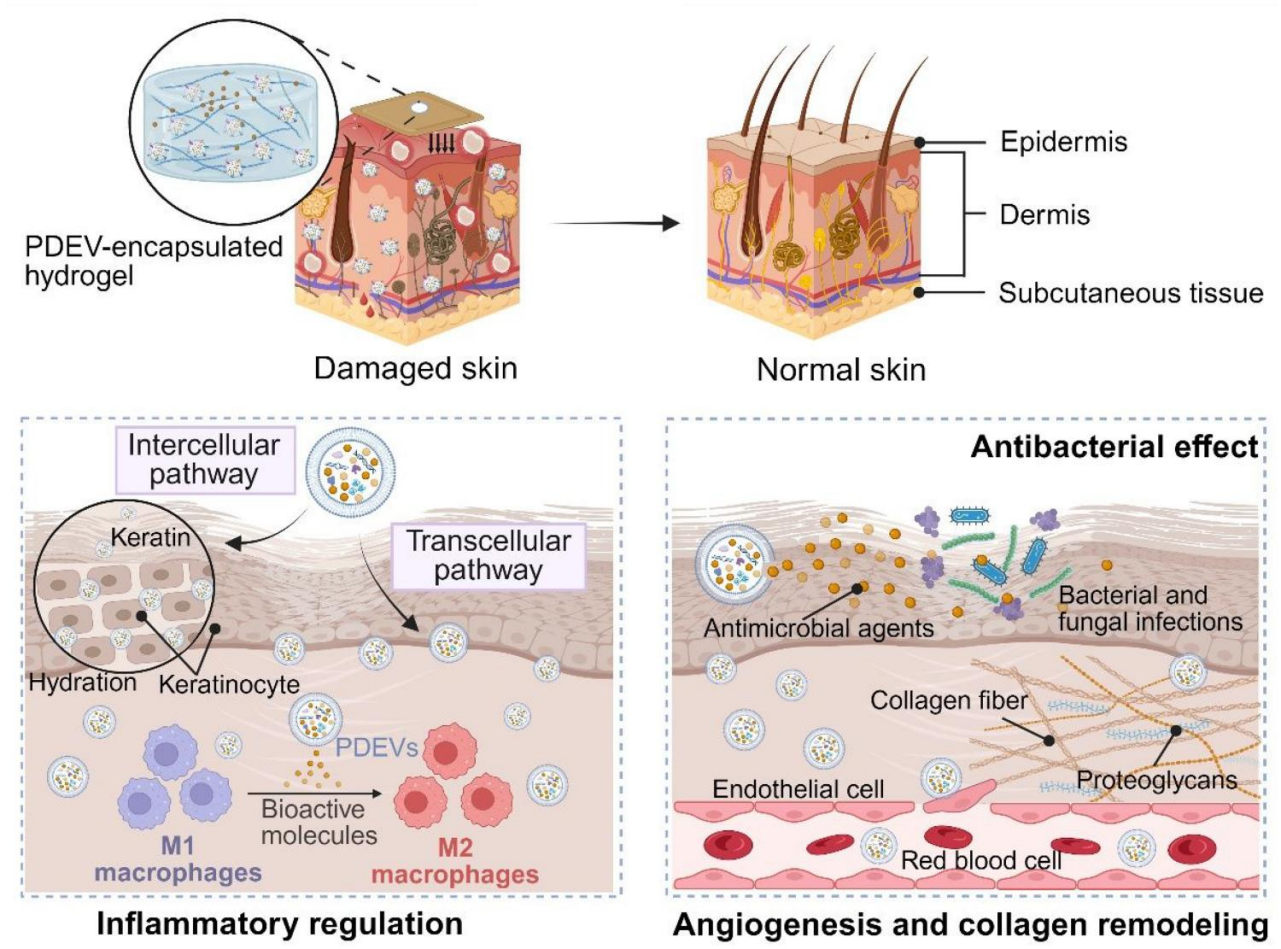
Plant-derived extracellular vesicles combined with hydrogel for treating skin-related diseases.

The PDEV-hydrogel combination exhibits significant potential for treating skin-related diseases, as illustrated in Figure 6. PDEVs derived from *Olea europaea* leaves were incorporated into a cross-linked hyaluronic acid and tannic acid hydrogel with strong UV-absorption properties. This combination synergistically alleviates photoaging damage through a dual ‘defense-repair’ mechanism: tannic acid in the hydrogel absorbs UV radiation, while PDEVs concurrently release active components (e.g. antioxidant polyphenols, miRNAs) to repair UV-induced cellular damage, promote collagen regeneration and suppress inflammatory-aging pathways [147]. Moreover, the combination of a wireless thermoelectric hydrogel with REDV peptide-modified ginseng-derived PDEVs activates the peroxisome proliferator-activated receptor signaling pathway, promoting wound re-epithelialization, orderly collagen remodeling, angiogenesis and the reconstruction of endogenous electric fields, thereby accelerating diabetic ulcer healing [137].

Plant-derived extracellular vesicles (PDEVs) effectively enhance drug penetration by entering the dermis via transcellular and intercellular pathways. The bioactive molecules carried by PDEVs promote macrophage polarization and modulate inflammatory responses, while simultaneously stimulating angiogenesis and collagen remodeling. Furthermore, PDEVs loaded with antimicrobial agents can effectively treat skin infections, thereby facilitating the restoration of damaged skin to its normal state. Created with BioRender.com.

### Vaccine development

PDEVs can be used to deliver mRNA vaccines, cancer vaccines and recombinant vaccine antigens, enabling vaccination via oral administration, intramuscular injection or nasal delivery [122, 172, 173]. When used for the oral delivery of mRNA and cancer vaccines, PDEVs not only protect the payload from degradation by enzymes and the acidic/alkaline environment in the gastrointestinal tract but also facilitate penetration across the intestinal epithelial barrier via endocytosis, demonstrating favorable absorption at the mucosal level [122, 172]. In one study, green fluorescent protein mRNA, Alexa Fluor 647-labeled HSP70 and recombinant hepatitis B surface antigen were loaded onto PDEVs derived from grapefruit (*Citrus* × *paradisi*) and mandarin orange (*Citrus reticulata*) using electroporation. Experimental results demonstrated that these antigen-loaded PDEVs were effectively delivered into cells, leading to the expression of functional proteins [173]. Furthermore, multiple SARS-CoV-2 mRNA vaccines can be loaded into and delivered to target cells by orange (*Citrus sinensis*) juice-derived PDEVs, where they are translated into proteins, thereby activating immune responses [123]. Animal studies further confirmed that mice administered the vaccines via multiple routes generated SARS-CoV-2-specific antibodies and cellular immune responses. Notably, oral and nasal administration also induced mucosal immune responses, producing specific IgA antibodies, which are critical for preventing viral invasion.

### Cardiovascular disorders

PDEV-based drug delivery systems demonstrate significant potential for treating cardiovascular diseases. For instance, *Morus alba* L. leaf-derived PDEVs, modified with cRGD, specifically target activated platelets at thrombus sites. This modification significantly increases the enrichment efficiency of urokinase-type plasminogen activator at the lesion site to 74.3%. Meanwhile, the natural flavonoid components of PDEVs scavenge ROS in the thrombotic microenvironment and promote macrophage conversion into the reparative M2 phenotype, thereby achieving dual therapeutic effects of thrombolysis and vascular repair [174]. In another study, successful electroporation loading of an antagomir targeting heart-apoptosis-associated piRNA into PDEVs derived from green tea demonstrates that these orally administered particles are absorbed through the gastrointestinal tract, enter systemic circulation and specifically target aortic lesion sites. By inhibiting phenotypic switching in vascular smooth muscle cells and downregulating matrix metalloproteinase-9 and myocyte enhancer factor 2D expression, the PDEVs effectively alleviate vascular dilation and elastic fiber fragmentation, significantly reducing aortic dissection incidence, while simultaneously improving murine survival rates [116]. The applications of PDEVs as drug delivery systems were compiled in Table 5.

**Table 5:** Applications of plant-derived extracellular vesicles as drug delivery systems.

Plant	Loading method	Modification	Loaded drug	Loading capacity/efficiency	Application	Model	Therapeutic efficacy	References
*Lycium barbarum* L.	Co-incubation	NA	Isoliquiritigenin	Loading capacity: 216 ± 31 μg/mg (based on protein content)	Spinal cord injury repair	Cell: neural stem cells and N9 microglial cells Animal: SD rats	*In vitro*: promoted neural differentiation, induced synaptic growth, inhibited inflammation and decreased ROS *In vivo*: improved motor function, reduced glial scarring, promoted nerve regeneration, decreased pro-inflammatory cytokines	[101]
Ginger	Ultrasonic incubation	NA	Curcumin	Loading capacity: 94.027 ± 0.094% Loading efficiency: 89.300 ± 0.344%	Ulcerative colitis	Animal: C57BL/6 mice with DSS-induced ulcerative colitis	*In vivo*: modulated inflammatory cytokines (TNF-α, IL-1β, IL-6, IL-10); regulated serum metabolites and gut flora; improved disease activity index, colon length, organ coefficients; reduced myeloperoxidase activity	[54]
*Rabdosia rubescens*	Extrusion	NA	Oridonin	Loading capacity: 9.21 ± 0.45% Loading efficiency: 76.4 ± 3.2%	Chemo-radiotherapy-induced oral mucositis	Cell: L929 fibroblast cells Animal: SD rats with oral mucositis	*In vitro*: enhanced L929 cell migration, antibacterial and biocompatibility *In vivo*: accelerated wound healing, reduced IL-1β, IL-6, TNF-α, promoted collagen deposition, targeted NLRP3 to reduce inflammation	[102]
Celery (*Apium graveolens* L.)	Co-incubation	NA	Doxorubicin (DOX)	Loading efficiency: 87.03%	Lung cancer	Cell: A549, PC3, C42B, HUVEC cell lines Animal: BALB/c nude mice with A549 xenograft tumors	*In vitro*: superior cytotoxicity and lower IC_50_ against A549 cells compared to Liposome-DOX *In vivo*: enhanced tumor growth inhibition; reduced systemic toxicity of DOX	[109]
Cucumber	Co-incubation	NA	Terbinafine	Loading capacity: 44.833 ± 0.23% Loading efficiency: 40.636 ± 0.38%	Cutaneous fungal infections	Microbial: candida albicans strains (SC5314, Yem13) Animal: BALB/c mice	*In vitro*: inhibited fungal growth and induced fungal membrane damage *In vivo*: reduced fungal load, decreased inflammation and promoted skin healing	[112]
Tomato fruit	Ultrasonication, freeze-thaw cycles, incubation at room temperature	NA	Calcitriol	Loading efficiency: 76.18%	Colorectal cancer	Cell: HCT116, HT29 colon cancer cells	*In vitro*: enhanced cytotoxicity (lower IC_50_ values compared to free calcitriol); reduced colony formation and migration; elevated ROS levels and induced apoptosis	[113]
Green tea	Electroporation	NA	Antagomir-HAAPIR complex	Loading capacity: approximately 100 ng/200 μg	Aortic dissection (AD)	Cell: HASMCs Animal: mouse AD model (Ang II/BAPN induced)	*In vitro*: inhibited HASMCs phenotypic switch *In vivo*: reduced AD incidence and aortic dilation; improved survival rate	[116]
*Sophora Flavescens*	Electroporation	NA	CX5461	Loading efficiency: 23%	Ulcerative colitis	Cell: RAW264.7 macrophages, Caco-2 enterocytes Animal: C57BL/6 mice with DSS-induced colitis	*In vitro*: inhibited M1 macrophage proliferation and promoted apoptosis; promoted M2 macrophage polarization; suppressed pro-inflammatory factors *In vivo*: alleviated colitis symptoms and weight loss; reduced colon shortening and tissue damage	[118]
Ginger	ExoFect transfection	Folic acid	siRNA (survivin)	Loading efficiency: approximately 80%	Antitumor	Cell: KB cells (human oral epidermoid carcinoma), HEK293, RAW 264.7 Animal: KB cell xenograft in nude mice	*In vitro*: knocked down survivin mRNA and exhibited low cytotoxicity *In vivo*: suppressed tumor growth; reduced survivin protein level	[120]
Orange (*Citrus sinensis*) juice	Osmotic shock-based loading	NA	SARS-CoV-2 mRNA	Loading capacity: 3.51 ± 1.09 ng/10^11^ PDEVs Loading efficiency: 72 ± 11%	Vaccine delivery	Cell: human macrophages (MV-4-11), HMEC-1, NHDF, PBMC Animal: BALB/c mice	*In vitro*: protected mRNA from RNase/SGF degradation; delivered mRNA to cells and translated into protein; activated lymphocytes *In vivo*: induced specific IgM, IgG, IgA antibodies, neutralizing antibodies and T-cell response via oral, intranasal and intramuscular routes	[123]
Ginger	Magnetic stirring	Tumor-targeting aptamer AS1411	Curcumin	Loading efficiency: 36.8%	Ovarian cancer	Cell: ID8 cells (mouse ovarian cancer cells) Animal: female BALB/c mice with ID8 tumor	*In vitro*: dose-dependent cytotoxicity in ID8 cells *In vivo*: suppressed tumor growth, reduced bacteria, decreased ROS	[124]
Grapefruit	Ultrasonication	Folic acid ligand	miR17	Loading efficiency: 86.2 ± 5.7%	Brain tumors (e.g., glioblastoma)	Cell: GL-26 brain tumor cells Animal: C57BL/6j mice with intracranial GL-26 tumors	*In vitro*: efficiently delivered miR17 to GL-26 cells and reduced MHC I expression *In vivo*: delayed brain tumor growth; prolonged survival; increased NK cell infiltration and decreased MHC I+ tumor cells	[131]
*Rhodiola rosea*	Ultrasonication, co-incubation	DSPE-PEG2000-pYEEIE	DOX	NA	Melanoma	Cell: A375, CCC-HPF-1	*In vitro*: enhanced cytotoxicity and cell uptake; G1 phase cell cycle arrest	[133]
Ginger	Ultrasonication	NA	Anti-TNF-α antibody	Loading capacity: 51.5%	Inflammatory bowel disease	Cell: HT29 cells and RAW 264.7 cells Animal: DSS-induced colitis in C57BL/6 mice	*In vitro*: protected INF from degradation, enhanced cellular uptake in HT29 cells *In vivo*: recovered body weight loss, reduced disease activity index, increased colon length and decreased TNF-α levels in colonic tissues	[138]
*Cucurbita moschata*	Co-incubation	EpCAM aptamers	ZIF-8 loaded with DOX	Loading capacity: 28%; Loading efficiency: 88%	Prostate cancer	Cell: PC-3, CHO, RAW 264.7 Animal: PC-3 xenograft mice	*In vitro*: enhanced cellular uptake and induced higher apoptosis; exhibited superior targeted cytotoxicity (IC_50_ was 1.84 µg/mL at 24 h) *In vivo*: inhibited tumor growth; targeted enrichment in tumor cells	[132]
Ginseng root	Electroporation	Neutrophil membrane engineering	miRNA 182-5p	NA	Alleviation of sepsis-induced acute lung injury (ALI)	Cell: MLE-12 cells and C166 cells Animal: C57BL/6J mice with LPS-induced ALI	*In vitro*: reduced oxidative stress, suppressed inflammatory cytokines, improved mitochondrial function *In vivo*: decreased lung wet/dry ratio, ROS, MPO and inflammatory cytokines, improved lung histopathology and inhibition of NOX4/Drp-1/NLRP3 pathway in mouse ALI model	[134]
Ginger	Electroporation	NA	tFNA-AMP-CPG	Loading efficiency: 76.18 ± 7.10%	Parkinson’s disease (PD)	Cell: RAW264.7, BV2, SHSY-5Y, RIN-14B; Animal: C57BL/6 mice (MPTP-induced PD model)	*In vitro*: increased 5-HT expression; reduced expression of IL-6, TNF-α in RAW264.7 and BV2 cells; inhibited apoptosis and reduced α-syn expression in SHSY-5Y cells *In vivo*: improved motor symptoms; normalized gut microbiota; reduced inflammatory cytokines	[169]
Mulberry (*Morus alba* L.) leaf	Ultrasonication	Modified with cyclic RGD peptides	Urokinase-type plasminogen activator	Loading efficiency: 87.8%	Venous thromboembolism	Cell: RAW264.7, HUVEC Animal: KM mice with femoral vein thrombosis	*In vitro*: scavenged ROS and promoted M1-to-M2 macrophage transition *In vivo*: dissolved blood clots, reduced ROS levels and repaired blood vessels	[174]
*Allium tuberosum*	Co-incubation	NA	Dexamethasone	Loading capacity: 216 ± 31 μg/mg	Neuroinflammation	Cell: BV-2 and MG-6 mouse microglial cells	*In vitro*: reduced LPS-induced production of NO, TNF-α and IL-6; upregulated expression of anti-inflammatory HO-1	[150]

Abbreviations: *5-HT*, 5-Hydroxytryptamine; *Drp-1*, dynamin-related protein 1; *DSPE*, 1,2-distearoyl-sn-glycero-3-phosphoethanolamine; *DSS*: dextran sulfate sodium; *EpCAM*, epithelial cell adhesion molecule; *HAAPIR*, heart-apoptosis-associated piRNA; *HASMCs*, human aortic smooth muscle cells; *HO-1*, heme oxygenase-1; *HUVEC*, human umbilical vein endothelial cells; *IC_50_*, half maximal inhibitory concentration; *IgA*, Immunoglobulin A; *IgG*, Immunoglobulin G; *IgM*, Immunoglobulin M; *IL-1β*, interleukin-1 beta; *IL-6*, interleukin-6; *LPS*, lipopolysaccharide; *MHC-I*, major histocompatibility complex class I; *MPTP*, 1-methyl-4-phenyl-1,2,3,6-tetrahydropyridine; *NLRP3*, nucleotide-binding domain and leucine-rich repeat-containing family, pyrin domain-containing 3; *NOX4*, NADPH oxidase 4; *PEG*, polyethylene glycol; *ROS*, reactive oxygen species; *SD*, Sprague-Dawley; *tFNA-AMP-CPG*, Antimicrobial peptide-functionalized tetrahedral framework nucleic acid complex; *TNF-α*, tumor necrosis factor-alpha; *ZIF-8*, zeolite imidazolate framework-8.

### Cosmetics and nutraceuticals

In cosmetics, PDEVs serve as carriers for active ingredients like acetyl hexapeptide-8 and resveratrol, thereby addressing challenges such as poor skin penetration, instability and limited bioavailability. PDEVs from *Leontopodium alpinum* facilitated acetyl hexapeptide-8 dermal penetration in both *in vivo* and *in vitro* models. In a separate zebrafish wrinkle model, this nanoformulation upregulated elastin (*eln1*) expression, suggesting anti-wrinkle potential that requires validation in mammals [29]. Similarly, encapsulation in *Leontopodium alpinum*-derived PDEVs enhanced the transdermal absorption and cellular uptake of resveratrol, leading to effective inhibition of cellular senescence while exerting potent ROS-scavenging and anti-inflammatory effects [175]. A recent study [176] subjected a topical formulation containing 2% *Malus domestica*-derived PDEVs using a battery of standardized *in vitro* tests for genotoxicity, cytotoxicity, corrosion, irritation and sensitization and validated its anti-inflammatory and anti-wrinkle efficacy in human clinical trials. The results demonstrated that *Malus domestica*-derived PDEVs were safe and effective active ingredients, significantly reducing skin erythema and improving wrinkle length, volume and roughness, thereby establishing their applicability for sensitive skin and long-term use. These findings substantiate the commercial development of PDEVs as delivery vehicles for bioactive ingredients.

**Figure 7:**
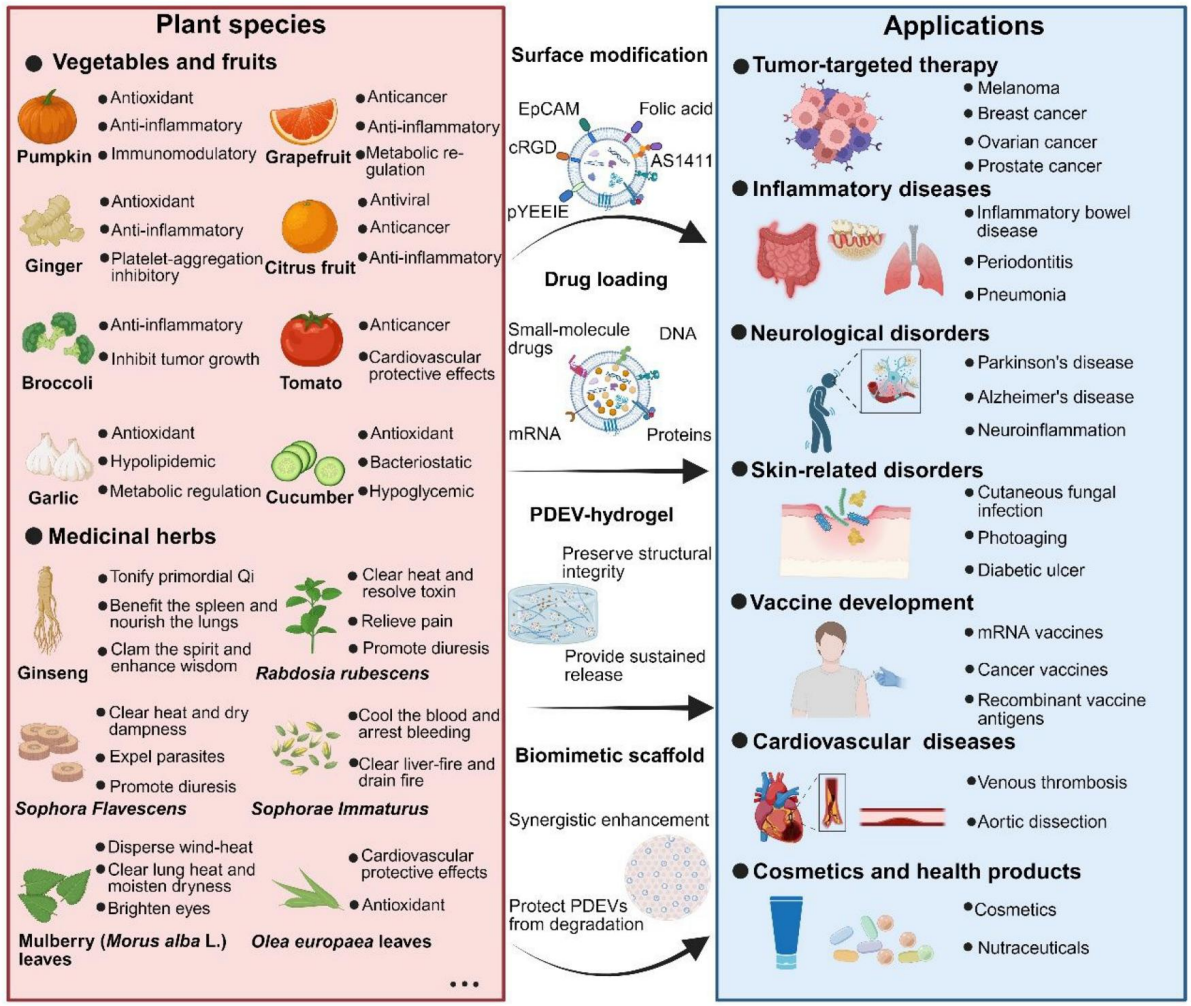
Plant-derived extracellular vesicles and therapeutic applications. Created with BioRender.com.

Edible plant-derived PDEVs are promising candidates for functional foods and dietary supplements. Their applications extend to food additives and functional beverages, conferring benefits for skin health and gut microbiome modulation, as well as metabolic and immune regulation. For instance, orange-derived PDEVs function as natural nanocarriers, significantly improving polyphenol stability and bioaccessibility to potentiate the antioxidant efficacy of functional beverages [177]. Similarly, PDEVs derived from ginger, lemon and tartary buckwheat promote the growth of beneficial bacteria (e.g. *Lactobacillus* spp.) and regulate microbial gene expression via miRNA cargo, stimulating short-chain fatty acid production and thereby enhancing intestinal barrier function [178]. In nutraceutical applications, strawberry- and citrus-derived PDEVs, which are rich in vitamin C and polyphenols, exhibit potent antioxidant activity beneficial for skin health [179]. Metabolically, grape- and ginger-derived PDEVs regulate blood glucose and lipid levels, suggesting utility in nutritional interventions for diabetes and obesity [26]. Furthermore, blueberry- and broccoli-derived PDEVs, characterized by specific anti-inflammatory mechanisms, show promise as therapeutics for chronic inflammatory conditions such as inflammatory bowel disease and arthritis [26]. Importantly, PDEVs offer safe, natural alternatives to synthetic additives, enhancing the clean-label profile of food products [178]. The sources and applications of PDEVs are illustrated in Figure 7.

## Challenges and approaches

### Quality control and scalable production

The diverse sources of PDEVs offer abundant and exploitable feedstock for large-scale production. However, achieving stable yields and controlling costs are severely hampered by inefficient purification, impurity carryover and low recovery yields. Moreover, the inherent diversity and heterogeneity of PDEV sources further complicate quality control during downstream processing. Studies have demonstrated significant inter-species variations in nanoparticle yield, size distribution, protein content and antioxidant activity, underscoring the challenges in standardizing production processes [180]. Even within the same species, drug loading efficiency is influenced by the growth stage and tissue type of the plant [181].

Currently, the commonly used isolation and purification methods primarily include UC, sucrose density gradient centrifugation, polyethylene glycol precipitation, ultrafiltration centrifugation, size-exclusion chromatography, immunoaffinity capture and microfluidic techniques [20, 182]. These methods rely on principles such as particle size variation, density gradient, surface protein labeling and hydrodynamic volume, combined with centrifugal force, molecular sieving effects or competitive binding of polymers to effectively separate PDEVs from impurities (e.g. cellular debris, protein) [20]. Despite demonstrating certain separation efficacy in laboratory studies, these methods still face significant challenges when scaled for production. Issues include the absence of standardized protocols, high equipment dependency and technical complexity. As shown in Table 6, the choice of isolation method significantly influences the physical properties of PDEVs, such as particle size and zeta potential, as well as final yield.

**Table 6:** Yield and physical characteristics of plant extracellular vesicles obtained by different isolation methods.

Plant	Source material	Extraction method	Particle characteristics	Particle concentration	Protein content	References
*Arabidopsis thaliana*	Callus culture	Ultracentrifugation	Size: 222.8 ± 36.5 nm Zeta potential: −23.8 ± 1.3 mV	1.8 × 10^10^ particles/g	NA	[34]
	leaf apoplastic fluid	Ultracentrifugation	Size: 283.6 ± 58.3 nm Zeta potential: −30.5 ± 2.2 mV	2.9 × 10^10^ particles/g	NA	[34]
*Curcuma longa* L.	Juice	Ultracentrifugation	Size: 183.2 ± 10.9 nm Zeta potential: −17.6 ± 1.19 mV	4.51 × 10^8^ 5.85 × 10^7^ particles/mL	0.125 ± 0.0162 mg/mg	[36]
Lane Late navel orange	Juice	Ultracentrifugation and sucrose density gradient centrifugation	Size: 154.5 ± 1.9 nm	6.33 × 10¹¹ particles/mL	210 ng/mL	[115]
Ginger	Juice	Differential centrifugation and commercial exosome isolation kits	Size: 163 ± 28 nm Zeta potential: −23.7 mV	2.1 × 10^10^ particles/mL	3010 ng/μL	[191]
Ginger	Juice	Ultracentrifugation and sucrose density gradient centrifugation	Size: 386.6 nm Zeta potential: −24.6 mV	NA	3.79 ± 0.27 mg/g	[192]
*Viburnum opulus*	Juice	Polymer precipitation and size-exclusion chromatography	Size: 45.36 nm Zeta potential: −2.87 mV	NA	1534 ± 97.78 μg/mL	[193]
*Solanum nigrum* L.	Juice	Sequential centrifugation and PEG precipitation	Size: 107 nm Zeta potential: −0.6 mV	8.1 × 10^9^ particles/mL	275.38 μg/mL	[183]

Therefore, the pretreatment of raw materials and the establishment of efficient separation and purification protocols are prerequisites for producing high-purity PDEVs. The diverse physical characteristics of plant sources necessitate distinct methodological approaches for PDEV isolation. For succulent berries, such as grapes [55], tomatoes [99] and black nightshade (*Solanum nigrum* L.) [183], juice is extracted by mechanical pressing. For fiber-rich botanical sources such as *Pueraria lobata* [184], turmeric (*Curcuma longa* L.) [36] and *Salvia miltiorrhiza* [79], homogenates require coarse filtration to remove fibrous debris before purification. For leaves and fruit peels, PDEVs are isolated from apoplastic fluid collected via infiltration-centrifugation [185, 186]. In contrast to extraction from intact plant organs, plant tissue culture-based PDEV production ensures greater standardization, as the strictly sterile environment virtually eliminates pathogen contamination. Currently, plant cell suspension cultures, callus, protoplasts, hairy roots and pollen cultures have been used to isolate PDEVs [17].

Optimizing separation and purification remains critical. Beyond conventional methods, innovative strategies aim to improve efficiency, reduce costs and enhance scalability. For instance, an integrated electrophoretic-dialysis technique enables the isolation of PDEVs from lemon juice without expensive instrumentation. Using 300 kDa dialysis membranes with electric field assistance, this method concentrated PDEVs in 2.5 h (compared with 4 h for UC) and yielded vesicles with comparable size distribution and morphology [187]. Additionally, the combination of pH adjustment to 4–5 and PEG6000 precipitation increased PDEV yield 5-fold and total phenolic content 3- to 4-fold, without compromising particle size stability or functional integrity [188]. Furthermore, enzymatic digestion with cellulase and pectin lyase reduced co-extraction of impurities from complex plant cell wall constituents, thereby improving extract purity and yield [189]. Zhao *et al*. [190] developed an enzymatic hydrolysis-UC technique for PDEV isolation from *Morinda officinalis*. Compared with traditional grinding, this approach achieved significantly higher yield, size homogeneity, protein content and endothelial uptake efficiency.

### Standardized characterization and quality assessment

When PDEVs are used for drug delivery, the diversity of drug-loading methods and engineered modifications introduces additional complexity to quality control, resulting in significant variations in physicochemical properties (e.g. size distribution, zeta potential, membrane integrity) and functional attributes (e.g. drug loading capacity, encapsulation efficiency, release kinetics). Therefore, establishing standardized characterization techniques and a multi-dimensional quality evaluation system is crucial.

As mentioned previously, current practices typically involve microscopy for morphological characterization of PDEVs, DLS and NTA for determining PDEV size distribution and various analytical techniques such as western blotting, fluorometric assays, gel electrophoresis, gene sequencing, liquid chromatography-tandem mass spectrometry and Fourier-transform infrared spectroscopy for compositional analysis of PDEVs [6, 191, 194]. However, PDEVs lack canonical biomarkers commonly present in ADEVs (e.g. CD63, CD9), and their surface molecular repertoire remains incompletely characterized [20]. Although HSP70, glyceraldehyde-3-phosphate dehydrogenase, S-adenosyl- homocysteinase, TET8, PEN1 and PEN3 are regarded as potential surface markers for PDEVs [20, 30], these molecules have not been widely used in routine identification of PDEVs. This discrepancy can be attributed to several fundamental limitations. First, these candidate proteins lack rigorous specificity validation [195]. Second, low marker conservation across plant species and inherent subpopulation specificity are further compounded by insufficient characterization of PDEV subtype composition and function [195, 196]. Third, varied isolation and purification methods have led to inconsistent PDEV definitions, precluding a unified framework for cross-study comparisons [38]. Fourth, commercial antibodies targeting putative PDEV markers remain scarce [38, 195]. Collectively, these limitations mean that PDEV identification and purity assessment often rely on conventional protein quantification, which inherently lacks the specificity to discriminate bona fide vesicular proteins from co-isolated contaminants.

However, concerted efforts to overcome these challenges are currently in progress. Systematic proteomic analysis has recently identified conserved marker families in PDEVs derived from *Arabidopsis thaliana* and *Brassica oleracea*, establishing the first molecular marker framework encompassing vacuolar-type ATPase subunits and plant-specific fasciclin-like arabinogalactan proteins [197]. This work provides crucial molecular insights that address long-standing challenges in PDEV research: consistent identification difficulties and poor comparability of findings, both stemming from a lack of standardized markers. Furthermore, introducing a Triton X-100 membrane disruption efficiency test as a key validation metric for sample purity assessment would further enhance the reliability of identification results [198].

The assessment of PDEV drug delivery systems is challenging due to the lack of standardized methods for evaluating drug-loading capacity. Steć *et al*. [146] addressed this gap by developing capillary electrophoresis and nanoplasmonic sensing techniques for process monitoring and quality control. Capillary electrophoresis enabled simultaneous detection of free DOX and DOX-loaded PDEVs, allowing real-time assessment of drug loading efficiency and leakage without prior separation. Meanwhile, nanoplasmonic sensing monitored drug-PDEV interactions, revealing DOX accumulation at the membrane interface and offering molecular insights into drug loading and carrier stability.

### Storage stability assurance

Since standardized protocols for the long-term storage of PDEVs remain underdeveloped, current research in the field predominantly relies on laboratory-prepared samples. During storage, the stability of PDEVs is influenced by multiple factors, including temperature, pH, oxidative stress, freeze-thaw cycles and buffer composition, generally manifesting as an increase in particle size and a decrease in protein content [199]. Storage at –20°C better preserves particle size stability but is associated with a pronounced reduction in protein concentration. In contrast, storage at 4°C helps maintain higher protein levels, yet often leads to vesicle aggregation [92]. PDEVs remain stable under neutral pH conditions (6.8–7.4) but prone to rupture and cargo leakage at acidic pH (<4.0) [199]. Therefore, maintaining appropriate temperature and pH is critical for preserving PDEV integrity during storage. For extended storage at 4°C (e.g. one month), the addition of preservatives stabilizes the system pH at approximately 5.0, effectively maintaining the vesicle size distribution and protein content [92]. When storing PDEVs at low temperatures (–20°C or –80°C), freeze-thaw cycles should be strictly limited. Studies demonstrate that leaf-derived PDEVs subjected to three freeze-thaw cycles exhibited a broadened size distribution, along with significant reductions in protein content and cellular uptake efficiency [92]. In comparison, *Kaempferia parviflora*-derived PDEVs maintained stability for 8 weeks at both –20°C and –80°C without freeze-thaw cycles [200].

For long-term preservation exceeding three months, lyophilization combined with an appropriate buffer system is critical. Without adequate protection, the lyophilization process poses a significant risk to the structural integrity and functional properties of PDEVs. For instance, lyophilization of pomegranate-derived PDEVs markedly reduced their yield, purity and membrane protein content (e.g. TET-8), thereby impairing anti-inflammatory and wound-healing bioactivities along with cellular uptake efficiency [201]. The incorporation of lyoprotectants, such as dimethyl sulfoxide (DMSO), offers a viable strategy. As demonstrated in a co-lyophilization study of orange-derived PDEVs with mRNA, 1% DMSO enabled stable room-temperature storage for one year while preserving vesicle morphology, mRNA integrity, and immunogenicity [202]. Other effective lyoprotectants for PDEVs include glycerol, ethylene glycol, sucrose and trehalose [30]. However, knowledge of optimal storage buffers for PDEVs remains limited. Although a combination of PBS-HAT buffer (containing human serum albumin and trehalose) and storage at –80°C is an optimized preservation strategy for ADEVs [203], its applicability to PDEVs remains unclear. Furthermore, developing preservation systems tailored to the characteristics of PDEVs remains a significant challenge for future research.

### Safety evaluation

Despite the superior safety profile of PDEVs compared to extracellular vesicles from other sources (e.g. animal, bacterial), their clinical application is hindered by the absence of systematic safety evaluation. Current research primarily focuses on acute and subacute toxicity, while in-depth assessments of long-term toxicity, *in vivo* metabolic fate and potential impacts within complex microenvironments are still insufficient. *In vitro* studies indicate that PDEVs exhibit no significant toxicity toward various human and mouse cell lines, even at high concentrations [111, 113, 174]. However, toxicity thresholds vary considerably depending on the plant source. PDEVs derived from *Allium tuberosum* significantly reduced the viability of mouse microglial cells at concentrations reaching 40 µg mL^−1^ [150]. In contrast, PDEVs from *Solanum nigrum* L. berries markedly inhibited the survival of mouse macrophages (viability < 4%) at concentrations exceeding 5 µg mL^−1^ [183]. Certain PDEVs demonstrate selective cytotoxicity toward specific cancer cells. For instance, PDEVs derived from *Viburnum opulus* exhibited approximately 7.5-fold greater toxicity toward human glioblastoma cells than toward normal cells [193]. Similarly, PDEVs from *Rhodiola rosea* suppressed the proliferation of human melanoma A375 cells in a concentration-dependent manner, an enhancement further amplified by pYEEIE modification, without affecting normal cells [133]. *In vivo* studies have shown that, at therapeutic doses, PDEV-based drug delivery systems do not induce significant pathological injury, functional impairment or abnormal immune-inflammatory responses in major organs, indicating favorable biosafety [174, 204]. Therefore, it is essential to establish a safe and effective therapeutic window for PDEVs based on their origin and intended therapeutic use.

Importantly, the diversification of PDEV applications across food, pharmaceutical and cosmetic sectors demands tailored safety assessment strategies. For functional foods, systematic evaluation of structural stability under digestive conditions, processing resilience and food matrix interactions is essential [26, 205]. Pharmaceutical development requires rigorous definition of pharmacokinetic behavior, targeting efficiency and interspecies cargo stability, complemented by metabolite monitoring standards to govern *in vivo* outcomes [12, 15]. Cosmetic applications necessitate focused assessment of dermal penetration, cellular internalization and biocompatibility, validated through physiologically relevant exposure models [28].

### Regulatory framework development

A foundational regulatory framework for PDEV-based drug delivery systems has been introduced in China, with its accompanying technical standards undergoing iterative refinement. In June 2025, the Center for Drug Evaluation of China’s National Medical Products Administration released draft guidance that, for the first time, classified extracellular vesicles as novel delivery system-based medicinal products under the regulatory framework for advanced therapy medicinal products. In August of the same year, the consortium standard (T/CIET 1720–2025) for PDEV drug delivery systems has been officially released, providing unified technical guidance to accelerate their industrialization. In the cosmetics sector, a consortium standard (T/SHRH 075–2025) for plant-derived vesicle cosmetic raw materials was concurrently released.

However, these standards are not mandatory, as the corresponding regulatory details and penalty provisions have yet to be established. Moreover, it remains unclear whether distinct vesicle-based platforms, such as ADEVs and PDEVs, will be subject to differential regulatory frameworks. The research and development of PDEV drug delivery systems still lacks consensus-based technical guidelines on a global scale [25]. Therefore, regulatory advancement depends on interdisciplinary and international collaboration to establish unified standards and accelerate clinical translation.

## Conclusion and perspectives

As discussed above, PDEVs serve as natural drug carriers, encapsulating various endogenous active molecules and loading exogenous therapeutic agents such as proteins, small molecules and nucleic acids. The loading methods govern encapsulation efficiency and subsequent cargo stability; thus, rational design of a PDEV-based delivery system requires a loading strategy tailored to the physicochemical properties of the therapeutic agent. For hydrophilic drugs, physical methods such as electroporation or ultrasonication are employed to generate transient pores in the vesicle membrane, enabling efficient loading; for hydrophobic drugs, the co-incubation method is preferred to achieve drug loading under mild conditions, thereby preserving vesicle structural integrity and drug activity to the greatest extent [12–14]. Emerging drug loading strategies include microfluidic-based approaches, which enable scalable loading of siRNA and other therapeutics through precise control of fluid mixing [125]. Light irradiation-based methods exploit the endogenous photosensitizers PDEVs to generate reactive oxygen species upon illumination, thereby reversibly increasing membrane permeability for drug encapsulation [126]. Consequently, the applicability of this strategy is governed by the presence of adequate photosensitizing components within the PDEVs. The osmotic shock-based loading strategy, combining cationic interactions with controlled osmotic stress, requires high osmotic tolerance of PDEV membranes but enables efficient delivery of nucleic acid therapeutics (e.g. mRNA) while protecting them from degradation [122, 123]. The loading capacity of PDEVs increases with compound lipophilicity [49, 78], suggesting that hydrophobic modification serves as an effective strategy to enhance drug encapsulation. Through strategies such as ligand conjugation and membrane fusion, surface modification improves the targeting specificity of PDEVs and facilitates their cellular uptake, thereby enabling precise drug delivery. Unlike widely investigated exogenous loading methods, endogenous loading strategies, including genetic engineering and osmotic pressure modulation, offer a novel approach to engineering vesicles without compromising their structural integrity and thus hold great promise for future research.

In addition, the development of composite drug delivery systems offers a viable approach for designing advanced regenerative biomaterials with complementary functionalities and synergistic therapeutic effects. For instance, the composite drug delivery system formed by integrating PDEVs with hydrogels, scaffolds and microneedles exhibits unique advantages for tissue repair and regeneration. Hydrogels and scaffolds effectively mimic the structure and function of the native extracellular matrix, providing a moist microenvironment conducive to wound healing while serving as drug reservoirs for sustained and controlled release [101, 147, 206]. In contrast, microneedles physically disrupt the skin barrier for the direct delivery of PDEVs to deep lesions [207, 208]. This facilitates potent, localized therapy with minimal off-target effects, positioning it as an ideal strategy for targeted intervention in deep tissue injuries. Unlike unimodal therapies, which often prove inadequate in complex pathological milieus, these platforms harness the pleiotropic effects of their components to enable synergistic regulation of inflammation, infection control and tissue proliferation. This integrated strategy holds particular promise for treating refractory defects that are unresponsive to conventional therapies.

Compared with ADEVs, clinical research on PDEVs remains limited, with only two candidates having completed Phase I clinical trials (NCT04879810 and NCT01668849) [16]. Despite the growing promise of PDEV-based systems in therapeutics and nutraceuticals, clinical translation is hindered by low isolation yield, limited long-term stability and the absence of standardized quality-control and safety standards [12, 18]. Accordingly, we propose a more operationalized clinical translation framework structured around five interconnected pillars, all of which still require systematic development: (i) quality control and scalable production, which entails optimizing preprocessing procedures and rigorously defining critical process parameters and batch release criteria; (ii) standardized characterization and quality assessment through an integrated multi-method analytical strategy; (iii) storage stability assurance, achieved by developing a robust lyoprotective buffer system; (iv) safety evaluation, encompassing comprehensive long-term toxicity and context-dependent safety assessments; (v) establishment of a differentiated regulatory framework attuned to the unique attributes of PDEVs. With respect to the first pillar, abiotic stress conditions can enhance PDEV yield and confer unique bioactivity, implying that tailoring the external environment represents a practical approach to generate PDEVs with desired functionalities. Indeed, PDEVs exhibit enhanced secretion and actively participate in stress responses under adverse conditions [209]. Beyond these five pillars, pharmacokinetic characterization constitutes a sixth critical dimension that merits explicit recognition as a prerequisite for clinical translation. Current research has largely focused on pharmacodynamic outcomes, while the pharmacokinetics of PDEVs remain poorly characterized. Several studies have employed *in vivo* tracing to preliminarily investigate their accumulation in major organs [104, 192]; nevertheless, critical properties, including metabolic stability, metabolite identification and excretion routes, have yet to be systematically clarified [104]. With regard to topical transdermal delivery, PDEVs have been shown to facilitate local drug penetration into the skin [112, 170]. However, the quantitative distribution profile across distinct skin layers (e.g. epidermis, dermis and hair follicles) still lacks systematic characterization.

Furthermore, with the integration of nanomedicine and synthetic biology, PDEVs are expected to evolve from natural nanoparticles into engineered, programmable intelligent delivery platforms. In line with this trend, hybrid vesicles serve as a promising example. Specifically, fusing PDEVs with engineered ADEVs combines the low immunogenicity of plant-derived components with the targeting capability of animal-derived ones. In a recent study, this strategy enabled targeted delivery to inflamed skin lesions via the CCR6-CCL20 chemotactic axis and exerted synergistic therapeutic effects superior to either component alone in psoriasis and atopic dermatitis models [135]. Similarly, the hybrid PDEV-liposome system exhibits enhancement in structural stability and delivery efficiency, along with functional complementarity in therapeutic efficacy [210]. Moreover, the development of intelligent responsive drug delivery systems based on PDEV enables precise spatiotemporal control over drug release behavior, thereby enhancing the therapeutic index and reducing adverse effects, representing a critical direction for future development.
